# Texting and Mobile Phone App Interventions for Improving Adherence to Preventive Behavior in Adolescents: A Systematic Review

**DOI:** 10.2196/mhealth.6837

**Published:** 2017-04-19

**Authors:** Sherif M Badawy, Lisa M Kuhns

**Affiliations:** ^1^ Ann & Robert H. Lurie Children's Hospital of Chicago Department of Pediatrics, Division of Hematology, Oncology, and Stem Cell Transplant Northwestern University Feinberg School of Medicine Chicago, IL United States; ^2^ Department of Pediatrics, Division of Hematology/Oncology Zagazig University Faculty of Medicine Zagazig Egypt; ^3^ Ann & Robert H. Lurie Children's Hospital of Chicago Department of Pediatrics, Division of Adolescent Medicine Northwestern University Feinberg School of Medicine Chicago, IL United States

**Keywords:** adolescent, text messaging, smartphone, mobile phone, mobile applications, medication adherence, behavior, prevention

## Abstract

**Background:**

Many preventable behaviors contribute to adolescent mortality and morbidity. Non-adherence to preventive measures represents a challenge and has been associated with worse health outcomes in this population. The widespread use of electronic communication technologies by adolescents, particularly the use of text messaging (short message service, SMS) and mobile phones, presents new opportunities to intervene on risk and preventive risk behavior, but little is known about their efficacy.

**Objective:**

This study aimed to systematically evaluate evidence for the efficacy of text messaging and mobile phone app interventions to improve adherence to preventive behavior among adolescents and describe intervention approaches to inform intervention development.

**Methods:**

This review covers literature published between 1995 and 2015. Searches included PubMed, Embase, CENTRAL, PsycINFO, CINAHL, INSPEC, Web of Science, Google Scholar, and additional databases. The search strategy sought articles on text messaging and mobile phone apps combined with adherence or compliance, and adolescents and youth. An additional hand search of related themes in the Journal of Medical Internet Research was also conducted. Two reviewers independently screened titles and abstracts, assessed full-text articles, and extracted data from articles that met inclusion criteria. Included studies reflect original research—experimental or preexperimental designs with text messaging or mobile phone app interventions—targeting adherence to preventive behavior among adolescents (12-24 years old). The preferred reporting items of systematic reviews and meta-analyses (PRISMA) guidelines were followed for reporting results, and findings were critically appraised against the Oxford Centre for Evidence-based Medicine criteria.

**Results:**

Of 1454 records, 19 met inclusion criteria, including text messaging (n=15) and mobile phone apps (n=4). Studies targeted clinic attendance, contraceptive use, oral health, physical activity and weight management, sun protection, human papillomavirus (HPV) vaccination, smoking cessation, and sexual health. Most studies were performed in the United States (47%, 9/19), included younger adolescents (63%, 12/19), and had sample size <100 (63%, 12/19). Although most studies were randomized controlled trials (RCTs; 58%, 11/19), only 5 followed an intent-to-treat analysis. Only 6 of 19 studies (32%) incorporated a theoretical framework in their design. Most studies reported good feasibility with high acceptability and satisfaction. About half of the included studies (42%, 8/19) demonstrated significant improvement in preventive behavior with moderate standardized mean differences. As early efforts in this field to establish feasibility and initial efficacy, most studies were low to moderate in quality. Studies varied in sample size and methods of preventive behavior adherence or outcome assessment, which prohibited performing a meta-analysis.

**Conclusions:**

Despite the promising feasibility and acceptability of text messaging and mobile phone apps in improving preventive behavior among adolescents, overall findings were modest in terms of efficacy. Further research evaluating the efficacy, effectiveness, and cost-effectiveness of these intervention approaches in promoting preventive behavior among adolescents is needed.

## Introduction

The burden of morbidity and mortality in adolescents worldwide is increasing [1-3], and the prevention of communicable and noncommunicable diseases, particularly those related to modifiable behavior, has been emphasized as a key component of adolescent health [4]. Many problem behaviors in adolescents, such as tobacco, alcohol, and other drug use; risky driving; and unsafe sex are preventable to a large extent [4], and their associated negative outcomes could be mitigated with preventive interventions. For adolescents, adherence to preventive measures represents a challenge in that the immediate and short-term benefits are often hard to comprehend and the long-term benefits may not be fully appreciated.

Several systematic reviews and meta-analyses have found positive effects of interventions to reduce risk behavior among adolescents and young adults, including tobacco use [5-7], alcohol misuse [8-11], drug use [12,13], risky driving [8], and unsafe sex [14]; as well as interventions to promote health behaviors, such as use of contraception to prevent pregnancy [15-17], human papilloma virus (HPV) vaccination [18], oral health and hygiene [19,20], and nutrition and exercise promotion [21-23]. The widespread use of electronic communication technologies by young people, particularly the use of mobile phones and other mobile devices [24-26], presents new opportunities to intervene on risk and preventive behavior. To our knowledge, no recent systematic reviews have been conducted on the effects of texting (short message service, SMS) and mobile phone apps—the most widely used technologies by young people—to improve prevent risk behavior and promote adherence to preventive health behavior in adolescents. The objective of this review was to systematically evaluate the evidence for the efficacy of texting and mobile phone app interventions in improving adherence to preventive behavior among adolescents and describe intervention approaches used in the included studies that would inform future intervention development.

## Methods

### Study Design

The protocol for this review was registered with the international prospective register of systematic reviews (PROSPERO; CRD42015025907) [27] and covered literature published between 1995 and 2015 with no language limits. The search strategy looked for all articles on texting, phones, mobile phone apps, and portable software combined with adherence or compliance, and search terms related to child, pediatric, adolescents, and youth. We intentionally used the Boolean search term “OR” instead of “AND” to capture the most comprehensive set of articles possible to which we applied our eligibility criteria. We followed the guidelines for the preferred reporting items for systematic reviews and meta-analyses (PRISMA) in the reporting of evidence across the studies reviewed herein [28]. In brief, a medical librarian conducted the literature search in the following sources: MEDLINE, Embase, CENTRAL, CINAHL, PsycINFO, Web of Science, Center for Review and Dissemination, INSPEC, Proquest Dissertations, Scopus, ClinicalTrials.gov, WHO Clinical Trials, Controlled-Trials.org, IEEE Explore, and Google Scholar (Multimedia Appendix 1). An additional hand search of related themes in the *Journal of Medical Internet Research* was also conducted. Two independent reviewers (SB and LK) assessed abstracts and articles against eligibility criteria and critically appraised the methodological quality using established criteria from the Oxford Centre for Evidence-based Medicine [29]. Disagreements were resolved by discussion or consultation with a colleague, if needed.

### Eligibility Criteria

Eligible studies were original research articles that included randomized controlled trials, quasi-experimental studies, or pilot pre-post studies of texting or mobile phone–based apps designed to improve adherence to preventive or prophylactic behavior in adolescents aged 12-24 years [30]. Adherence was defined as “the extent to which a person’s behavior coincides with medical or health advice” [31,32]. Therefore, the term “adherence” included both prescribed medications and scheduled clinic appointments [31-33]. To be included in this review, the studies had to report at least one primary or secondary outcome related to adherence to preventive behavior. Studies focused on parents rather than on adolescents, disease monitoring without intervention, or use of other forms of technology (ie, other than mobile phone apps or texting) were excluded.

### Data Synthesis

We used a standardized form for data extraction. Data items in the extraction form included the following: first author’s name; publication year; country; aim of the study; participants’ age and sex; study design and setting; sample size; selection criteria; duration of intervention and follow-up; retention rate; components of study intervention (texting or mobile phone apps) and comparator (if applicable); adherence measures and outcomes; other related outcome; and theoretical framework. Data were analyzed and summarized qualitatively and quantitatively. Standardized mean differences (SMD) with 95% CIs were calculated—using means and standard deviations, pre- and postvalues, or frequencies of outcomes—to evaluate the efficacy of texting or mobile phone–based apps in improving adherence to preventive behavior and related outcomes [34]. Data were analyzed using StataCorp (2013, StataCorp LP).

## Results

### Literature Search

**Figure 1:**
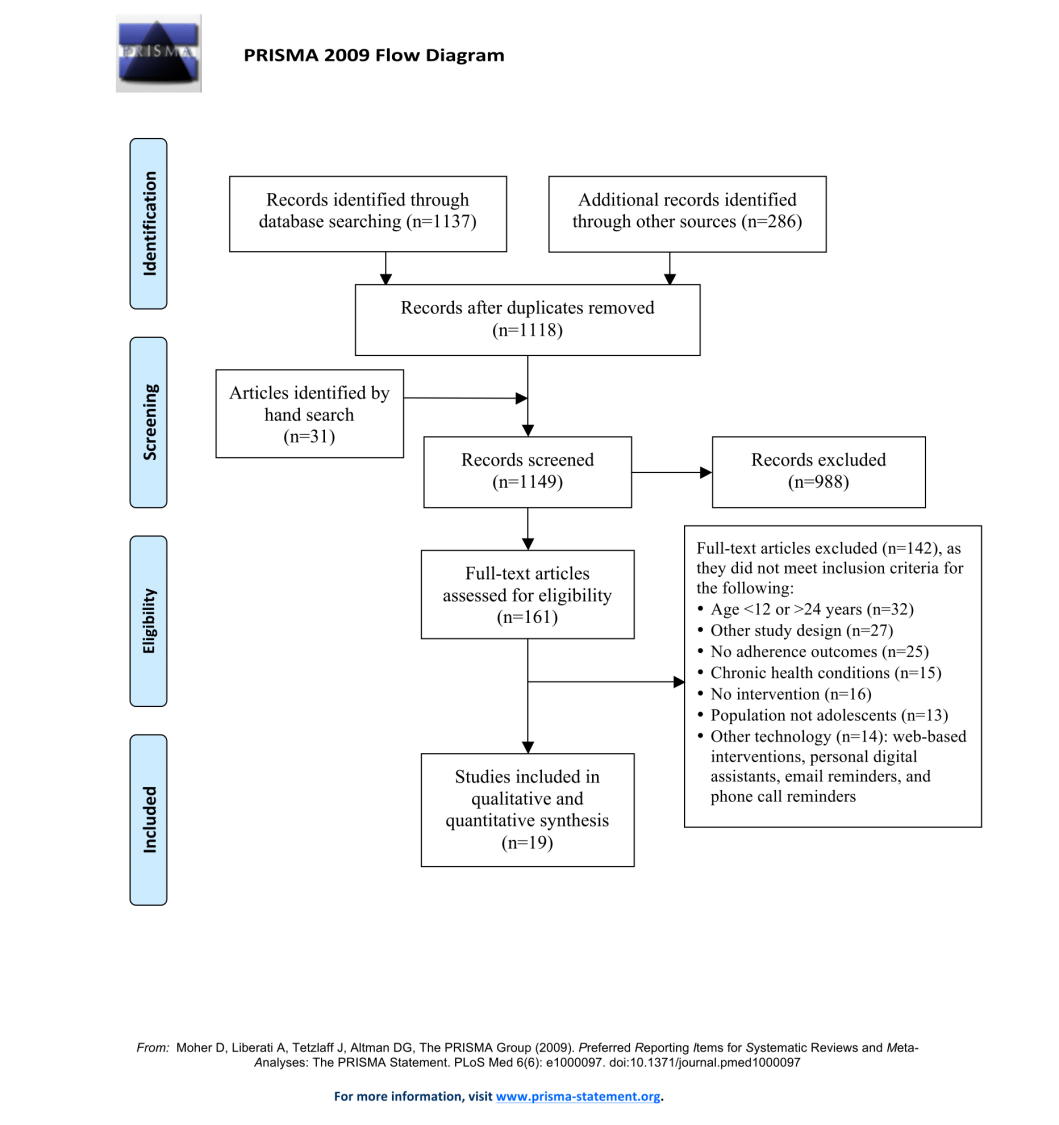
Flow of studies through the review according to the preferred reporting items for systematic reviews and meta-analyses (PRISMA) guidelines.

The literature search identified 1454 references (Figure 1), and 161 full articles were retrieved. Nineteen articles met all inclusion criteria [35-53]. Most (n=15) included texting interventions [35-41,43-45,47-49,52,53], and only 4 studies had mobile phone–based app interventions [42,46,50,51]. The primary aim of the interventions was to improve adherence to clinic attendance (primary care, gynecology, mental health) [35,36], contraception use [37-39], oral health and hygiene in orthodontic patients [46,47], physical activity and weight management [48-51], sun-protective measures [52], and HPV vaccination [53]; or to reduce risky behavior, including unsafe sex [40-42], smoking [45], and alcohol misuse [43,44].

### Description of Included Studies

Table 1 summarizes study characteristics (also see Multimedia Appendix 2). Nine were conducted in the United States [36-41,47,50,53], 3 in Switzerland [35,44,45], 2 in Hong Kong [48,49], and 1 each in Colombia [42], Wales [43], Italy[46], New Zealand [51], and Germany [52]. Most (n=9) were conducted in a clinic setting [35-39,46,47,53], 3 in a university [41-43], 2 in vocational schools [44,45], 1 in secondary schools [48], 1 in an emergency department [40], 1 in a clinic and summer camp [52], 1 in both a clinic and Web-based environment [50], and 1 at participants’ home [51]. Most (n=12) enrolled younger adolescents (age ≥12 and < 18 years) [35,36,41,45-53], 6 enrolled older adolescents (age ≥18 and <24 years) [37,38,40,42-44], and 1 reported only an age range of 13-21 years [39]. Fourteen studies indicated regular or mobile phone ownership or access as 1 of the eligibility criteria [35-40,42,44-48,51,52], 2 included patients with mobile phones [41,50], and 3 were not explicitly stated or reported [43,49,53]. Sample size ranged from 26 to 999, with a median of 78 and a mean of 232 participants per study; 12 enrolled < 100 [36,38,40,41,43,46-52], and 7 had ≥100 participants [35,37,39,42,44,52,53]. Participants’ race or ethnicity varied: majority were white in 5 [38,44,45,50,51], black in 3 [39-41], Asian in 2 [48,49], Latino in 1 [42], black and Latino in 2 [36,37], and not reported in 6 studies [35,43,46,47,52,53]. Only 6 studies incorporated or were informed by a clear theoretical framework for their intervention effects, including Transtheoretical Model [37], Geser’s Sociological Framework [39], Health Belief Model and Information Motivation Behavior Model [40], Health Action Process Approach [45], Stages of Motivational Readiness for Change Model [48], Addiction Treatment Model [50], or utilized specific effective self-regulatory behavior change techniques without an identified theoretical model [51].

**Table 1:** Summary of included studies focused on improving adherence to preventive measures in adolescents.

Source (country)	Aim of intervention	Study design (setting)	Age in years, (sex)	Group (n)	Tech	Follow-up	Retention rate	Grade
Narring et al [35] (Switzerland)	Improve attendance in clinic	RCT (randomized controlled trial), investigator-blinded (multidisciplinary youth clinic: primary care, gynecological and mental)	Intervention^a^: 17.7 (2.8) (75.9% Female) Control^a^: 17.7 (3) (75.4% Female)	Intervention (469) Control (530)	Text messages	6 months	Intervention: 462 (98.5%) Control: 529 (99.8%)	Moderate
Branson et al[36] (United States)	Improve attendance in mental health clinic	Quasi-experimental pilot study (large hospital mental health clinic)	Intervention^a^: 15.4 (1.3) (58% Female) Control^a^: 14.8 (1.5) (42% Female)	Intervention (24) Control (24)	Text messages	3 months	Intervention: 24 (100%) Historical Control: N/A^d^	Moderate
Castano et al [37] (United States)	Improve continuation of OCPs (oral contraceptive pills)	RCT, investigator-blinded (urban family planning health center)	Intervention^a^: 20.8 (2.5) Control^a^: 20.4 (2.7) All females	Intervention (480) Control (482)	Text messages	6 months	Intervention: 346 (72.1%) Control: 337 (69.9%)	Moderate
Hou et al[38] (United States)	Increase adherence to OCPs	RCT, investigator-blinded (Planned Parenthood League clinic)	Intervention^b^: 22 (18-31) Control^b^: 22 (18-30) All females	Intervention (41) Control (41)	Text messages	3 months	Intervention: 36 (88%) Control: 37 (90%)	Moderate
Trent et al [39] (United States)	Improving Depo-Provera appointment attendance	RCT, nonblinded (large urban academic General Pediatric and Adolescent Medicine Practice)	Intervention^b^: 13-21 Control^b^: 13-21 All females	Intervention (50) Control (50)	Text messages	12 months	Intervention: 33 (66%) Control: 36 (72%)	High
Suffoletto et al [40] (United States)	Reduce sex risk behavior among at-risk young adult F^g^ discharged from emergency department	RCT, nonblinded (urban tertiary hospital emergency department)	Intervention^a^: 22 (2) Control^a^: 21 (2) All females	Intervention (23) Control (29)	Text messages	3 months	Intervention: 15 (65%) Control: 21 (72%)	Low
Cornelius et al [41] (United States)	Improve HIV knowledge and attitudes toward condoms among African American adolescents	Pre-post single-arm pilot study (university)	Intervention^a^: 15.4 (1.7) (52.5% Female)	Intervention (40)	Text messages	3 months	Intervention: 36 (90%)	Low
Lopez et al [42] (Colombia)	Provide sexual education and improve knowledge about the sexual risk factors	Pre-post single-arm pilot study (university)	Intervention^a^: 21 (3.6) (53% Female)	Intervention (127)	App	6 months	Intervention: 58 (45.7%)	Very low
Moore et al [43] (Wales)	Reduce future alcohol consumption based on data of past alcohol expenditure	RCT, nonblinded (university)	Student^a^: 22 (3.7) Nonstudent^a^: 38.5 (14.3) Overall (33% Female)	Intervention (40): Student (21); nonstudent (19) Control (38): Student (16); nonstudent (22)	Text messages	2 months	Intervention: Student 20 (95%); nonstudent 18 (95%) Control: Student 14 (88%); nonstudent 22 (100%)	Low
Haug et al [44] (Switzerland)	Reduce alcohol binge or problem drinking	Pre-post single-arm pilot study (vocational school)	Intervention “participants”^a^: 18.0 (2.4) (24.5% Female) Control “nonparticipants”^a^: 17.8 (1.7) (19.5% Female)	Intervention (364) Control (113)	Text messages	3 months	Intervention: 280 (76.9%) Control: 87 (77.0%)	Moderate
Haug et al [45] (Switzerland)	Increase smoking cessation and reduce cigarettes consumption	Two-arm cluster RCT, assessor-blinded (vocational school)	Intervention^a^: 18.2 (2.4) (48.7% Female) Control^a^: 18.3 (2.2) (55.1% Female)	Intervention (372) Control (383)	Text messages	6 months	Intervention: 287 (77.2%) Control: 272 (71.0%)	High
Zotti et al [46] (Italy)	Improve oral hygiene adherence and oral health	RCT, assessor-blinded (orthodontic clinic in a university hospital)	Intervention^a^: 14.1 (58% Female) Control^a^: 13.6 (58% Female)	App (40) Control (40)	App	12 months	Intervention: 40 (100%) Control: 40 (100%)	Low
Bowen et al [47] (United States)	Improve adherence to oral hygiene and reduce plaque formation	RCT, patient-blinded (orthodontic clinic in a university hospital)	Intervention^a^: 15.5 (60% Female) Control^a^: 14.6 (56% Female)	Intervention (25) Control (25)	Text messages	3 months	Intervention: 19 (76%) Control: 21 (84%)	Low
Lau et al [48] (Hong Kong)	Promote physical activity in school age children	Quasi-experimental (secondary schools)	Intervention^a^: 12.3 (0.9) (68% Female) Control^a^: 13.3 (1.1) (63% Female)	Intervention (38) Control (40)	Text messages	2 months	Intervention: 38 (100%) Control: 40 (100%)	Low
Abraham et al [49] (Hong Kong)	Improve weight management	RCT, investigator-blinded (pediatric obesity clinic of a tertiary care hospital)	Intervention “Internet”^b^: 14.9, 13.7-16.2 (44% Female) Intervention “sLMP”^b^: 14.1, 13.5-15.3 (38% Female) Cnotrol^b^: 14.3, 13.5-16 (38% Female)	Intervention “Internet” (16) Intervention “sLMP” (16) Control (16)	Text messages	6 months	Intervention “Internet”: 16 (100%) Intervention “sLMP”: 16 (100%) Control: 16 (100%)	Low
Pretlow et al [50] (United States)	Improve weight management	Pre-post single arm pilot study (university hospital clinic and on the Internet)	Intervention^a^: 16 (0.43) (65% Female)	Intervention (43)	App	5 months	Intervention: 27 (63%)	Moderate
Direito et al [51] (New Zealand)	Improve fitness in insufficiently active healthy young people	Three-arm, parallel, RCT, nonblinded (participants home)	Intervention “immersive”^a^: 15.78 (1.11) (53% Female) Intervention “nonimmersive”^a^: 15.69 (1.04) (63% Female) Control^a^: 15.55 (1.32) (56% Female)	Intervention “immersive” (17) Intervention “non immersive” (16) Control (18)	App	2 months	Intervention “immersive”: 17 (100%) Intervention “non immersive”: 15 (94%) Control: 17/18 (94%)	Moderate
Sachse et al [52] (Germany)	Improve sun-protection knowledge and behavior	Pre-post single-arm pilot study (summer cap/clinic)	Intervention^a^: 16.1 y, 13-22 (27% Female)	Intervention (26)	Text messages	2 months	Intervention: 19 (73%)	Very low
Matheson et al [53] (United States)	Increase HPV (human papillomavirus) vaccination series completion rate	Pilot Quality Improvement project (urban pediatric clinic)	Intervention^a^: 12 (62% Female) Interested^a,c^: 14 (64% Female) Control^a,c^: 14 (42% Female)	Intervention (37) Interested^c^ (43) Control^c^ (232)	Text messages	8 months	Intervention: data not reported	Low

^a^Age reported as mean (SD, standard deviation).

^b^Age reported as median ( range, minimum-maximum).

^c^Interested group included patients or parents enrolled in the project during their clinic visit who did not complete their opt-in process to receive text message reminders after leaving clinic; historical control included all patients who initiated HPV vaccination series during study period, but were either not offered or declined to participate in the project.

^d^N/A: not applicable.

### Studies Methodological Quality

Most (n=11) were RCTs [35,37-40,43.45-47,49,51], 6 pre-post pilot design [41,42,44,50,52,53], and 2 quasi-experimental studies [36,48]. Of the RCTs, 7 were single-blinded—investigator (n=4), assessor (n=2), and patient (n=1) [35,37,38,45-47,49]; 4 nonblinded [39,40,43,51]; and none double-blinded. Overall, the quality of the included studies was low to moderate (Table 1). Details of allocation concealment and study blinding were inadequately reported for most studies. About half of the RCTs (n=5) used intention-to-treat analysis [35,37,38,45,51]. Almost all (n=18) reported retention rates, which differed across studies from <80% in 8 [37,38,40,42,44,45,50,52], ≥80 to <100% in 6 [35,38,41,43,47,51], 100% in 4 [36,46,48,49], and not reported in 1 study [53]. The duration of the interventions ranged from 2 to 12 months as follows: 2-3 months (n=11) [36-38,40,41,43,44,47,48,51,52], 5-8 months (n=6) [35,42,45,49,50,53], and 12 months (n=2) [39,46]. Only 2 studies extended follow-up for 2 [37] and 3 months [45] after the completion of active intervention.

### Description of Texting Interventions

Most (n=10) included texting as the only intervention [35-40,43,45,47,53], whereas 5 included additional components [41,44,48,49,52]: in-person training sessions in 2 [41,52], Web-based program in 2 [44,48], Internet-based curriculum in 1 [49], and a facilitator or a coach in 1 study [41]. Texting interventions varied in frequency, message content, and directionality of messages (also see Multimedia Appendix 3). Text reminders for frequent behaviors were sent once daily in 7 [37,38,41,43,45,48,52], and once or twice weekly in 5 studies [40,44,45,47,49]. Appointment reminders were sent at differing frequencies, including 1 day in 2 [35,36] or daily for 3 days before in 1 [39], or 7 days before, after and on the scheduled date in 1 study [53]. Text reminders were customized to the patient’s personal preferences in terms of scheduling (ie, day or time) in 5 studies [36-39.53], content in 8 [40,41,43-45,48,49,52], or both in 1 study [35]. Text-reminder directionality was 1-way in 7 [36-38,47,48,52,53] and 2-way in 8 [35,39-41,43-45,49], with emotion icon (emoji) response in 1 [49] and according to a sophisticated tailored algorithm in 3 studies [40,44,45]. All text reminders were sent to adolescents or young adults, not their parents. Text messages were also a tool for education in 7 [37,39,41,47,48,52], positive reinforcement or personalized feedback in 5 [40,44,45,48,49], goal setting in 3 [40,48,49], and addressing barriers in 1 study [48]. Most (n=9) provided incentives or a reward system for patient engagement [36,37,39-41,43-45,48]. One included a virtual friend “Jackie” who was part of all messages to build rapport with participants and provide more social support [48], and none included an explicit motivational approach or targeted social support network.

### Description of Mobile Phone App Interventions

In terms of the mobile phone platforms (also see Multimedia Appendix 4), 2 used existing commonly used mobile phone app platforms, “WhatsApp” [46] and “Zombies, Run! 5K Training app” or “Get Running-Couch to 5k app” [51], whereas 2 included apps developed specifically for the study [42,50]. Zotti et al [46] created an anonymous study group “Brush Game” on the app where patients shared 2 photographs of themselves weekly (“selfies”), participated in chat room conversations, shared materials related to oral hygiene, and viewed a weekly ranking of the top 5 participants based on chat room participation and oral hygiene outcomes. In contrast, Direito et al had participants randomized to either an immersive app “Zombies, Run! 5K Training” with a game-themed design embedded with a story where participants were trained to collect supplies and protect a town from zombies, or a nonimmersive app “Get Running-Couch to 5k” [51]. Both apps consisted of a fully automated 8-week training designed to improve fitness and ability to run 5 km, provided information on running and audio instructions on how to perform the training components, and tracked and displayed participant’s progress throughout the program; and included the ability to work out with music on the device’s library and links to associated websites to interact with other users [51]. Participants were encouraged to use their app 3 times per week and work their way through each of the workouts, but no cointerventions (ie, emails, phone calls, text message) or prompts to use the app were utilized [51]. On the contrary, Lopez et al developed a native app “DoctorChat” as an intervention that allowed participants to submit questions on different sexual and reproductive health issues through their mobile phones, and to receive personalized, accurate, and informative responses from health care professionals and experts in the field [42]. Moreover, Pretlow et al [50] utilized a multifunction app for weight management, in addition to group meetings and personalized coaching. The app included different capabilities such as progress reports, peer support, coping skills and self-management toolbox, fun activity ideas for distraction, and mentor communication [50].

### Intervention Effects on Adherence to Preventive Behavior and Other Outcomes

Most (n=12) reported clinical effects related to adherence to preventive behavior [40-51], 9 measured actual adherence to preventive behaviors [35-40.46,50,53], and 5 described other outcomes as well, including knowledge gain in 3 [41,42,52], motivational readiness in 1 [48], and change in self-management skills in 1 study [50] (also see Multimedia Appendices 5 and 6). Adherence to preventive behavior was evaluated by clinic attendance in 4 [35,36,39,53], self-report of adherence in 3 [37,40,50], self-report and electronic device to monitor adherence in 1 [38], and electronic direct observation of adherence behavior (self-photographs or selfies) in 1 study [46]. All the included studies provided enough information to calculate standardized outcomes (ie, effect sizes *d* or SMDs), except 1 [42]. At the end of the active intervention period, about half (n=8) of the studies reported significant improvement in adherence to preventive behavior and other related outcomes with moderate SMDs [36,37,44-47,52,53]. Table 2 summarizes SMDs for all included studies. Several studies reported findings for efficacy as well as usability and feasibility. Most (n=11) reported high levels of satisfaction and acceptability of study interventions [36-42,48-50,52].

**Table 2:** Effect sizes for the main outcomes of included studies.

Source (intervention)	Study outcomes	Effect size, *d* (95% CI)^a^
Narring et al [35] (Texting)	Attendance to all clinics	0.15 (–0.03 to 0.33)
	Attendance to primary care clinic	0.06 (–0.16 to 0.27)
	Attendance to gynecological clinic	0.32 (–0.10 to 0.74)
	Attendance to mental health clinic	0.32 (–0.21 to 0.85)
	Attendance to mental health clinic	0.32 (–0.21 to 0.85)
Branson et al [36] (Texting)	Attendance to mental health clinic	0.67 (0.09-1.25)^h^
Castano et al [37] (Texting)	Continued use of OCPs^b^ at follow-up: overall	0.23 (0.06-0.40)^h^
	Continued use of OCPs at ≤187 days	0.52 (0.19-0.86)^h^
	Continued use of OCPs at ≥188 days	0.13 (–0.07 to 0.33)
Hou et al [38] (Texting)	Decreased OCP doses miss rate: all participants	0.09 (–0.34 to 0.53)
	Decreased OCP doses miss rate: 3 cycles complete	0.13 (–0.30 to 0.57)
Trent et al [39] (Texting)	Depo-Provera on-time visit attendance: cycle 1	0.29 (–0.19 to 0.77)
	Depo-Provera on-time visit attendance: cycle 2	0.77 (–0.35 to 0.69)
	Depo-Provera on-time visit attendance: cycle 3	0.01 (–0.57 to 0.60)
Suffoletto et al [40] (Texting)	Condom use with last sexual intercourse	0.32 (–0.29 to 0.93)
	Always condom use in last 28 days	0.29 (–0.38 to 0.95)
	Drug or alcohol use before last sex	0.23 (–0.53 to 0.99)
	Any unprotected sex with concurrent alcohol use in last 28 days	0.58 (–0.41 to 1.57)
Cornelius et al [41] (Texting)	HIV knowledge	0.42 (–0.03 to 0.86)
	Attitudes toward condoms	0.08 (–0.36 to 0.52)
	Reduction in risky behavior: intercourse	0.17 (–0.27 to 0.60)
	Reduction in risky behavior: illegal drugs	0.41 (–0.03 to 0.86)
Moore et al [43] (Texting)	Decrease alcohold use in students	0.00 (–0.65 to 0.65)
	Decrease alcohol use in nonstudents	0.13 (–0.48 to 0.75)
Haug et al [44] (Texting)	Reduction in RSOD^c^ in persons with ≥1 occasion in the last month	0.22 (0.01- 0.42)^h^
	Reduction in RSOD in persons with >2 occasions in the last month	0.16 (–0.02 to 0.35)	
	Reduction in number of standard drinks in a typical week	0.14 (–0.02 to 0.31)
	Reduction in the maximum number of drinks on an RSOD occasion	0.08 (–0.09 to 0.24)
	Reduction in having one or more alcohol-related problems	0.24 (–0.01 to 0.48)
Haug et al [45] (Texting)	7-day smoking abstinence at 6 months: total sample	0.16 (–0.13 to 0.46)
	7-day smoking abstinence at 6 months: occasional smokers	0.25 (–0.21 to 0.71)
	7-day smoking abstinence at 6 months: daily smokers	0.15 (–0.28 to 0.59)
	4-week smoking abstinence at 6 months: total sample	0.08 (–0.31 to 0.47)
	4-week smoking abstinence at 6 months: occasional smokers	0.38 (–0.27 to 1.03)
	4-week smoking abstinence at 6 months: daily smokers	0.39 (–0.21 to 0.98)
	Reduction in cigarette consumption at 6 months: total sample	0.33 (0.16-0.50)^h^
	Reduction in cigarette consumption at 6 months: occasional smokers	0.36 (0.02-0.71)^h^
	Reduction in cigarette consumption at 6 months: daily smokers	0.20 (0.01-0.39)^h^
	Smoking quit attempts at 6 months: total sample	0.17 (–0.02-0.36)
	Smoking quit attempts at 6 months: occasional smokers	1.09 (0.65-1.52)^h^
	Smoking quit attempts at 6 months: daily smokers	0.06 (–0.16 to 0.29)
Zotti et al [46] (App)^d^	Gingival index at 6 months	0.56 (0.12-1.01)^h^
	Gingival index at 9 months	1.04 (0.57-1.51)^h^
	Gingival index at 12 months	1.43 (0.94-1.92)^h^
	Plaque index at 6 months	0.73 (0.28-1.18)^h^
	Plaque index at 9 months	1.50 (1.00-2.00)^h^
	Plaque index at 12 months	1.40 (0.91-1.89)^h^
	Visible white spots at 9 months	0.67 (0.04-1.30)^h^
	Visible white spots at 12 months	0.63 (0.06-1.20)^h^
Bowen et al [47] (Texting)	Plaque coverage reduction at 4 weeks	1.62 (0.98-2.26)^h^
	Plaque coverage reduction at 12 weeks	2.40 (1.67-3.12)^h^
Lau et al [48] (Texting)	Self-report of physical activity	0.31 (–0.14 to 0.76)
Abraham et al [49] (Texting)	Reduction in BMI (body mass index )	0.09 (–0.61 to 0.78)
Pretlow et al [50] (App)^e^	Reduction in percent over-BMI in males	0.17 (–0.55 to 0.88)
	Reduction in percent over-BMI in females	0.08 (–0.44 to 0.61)
Direito et al [51] (App)	Time to complete 1-mile walk or run using an immersive app	–0.238 (–0.9 to 0.43)
	Time to complete 1-mile walk or run using a nonimmersive app	–0.14 (–0.81 to 0.54)
Sachse et al [52] (Texting)	Understanding of the meaning of UV^f^-index	1.49 (0.61-2.37)^h^
	Naming ≥3 of ABCDE (ie, asymmetry, border, color, diameter, and evolution) mnemonic for skin self-exam	1.40 (0.19-2.61)^h^
	Knowing that it takes hours to recognize sunburns	0.51 (–0.24 to 1.26)
Matheson et al [53] (Texting)	HPV^g^ vaccine dose 2	1.10 (0.67-1.52)^h^
	HPV vaccine dose 2 on-time	0.46 (0.06-0.86)^h^
	HPV vaccine dose 3	0.70 (0.14-1.27)^h^
	HPV vaccine dose 3 on-time	0.91 (0.24-1.58)^h^
		

^a^Positive effect size value means improvement in a study outcome, while a negative one means worsening outcome.

^b^OCPs: oral contraceptive pills.

^c^RSOD: risky single-occasion drinking.

^d^Gingival index score (0-3): 0 being normal gingiva and 3 having severe inflammation and edema, with spontaneous bleeding; plaque index score (0-3): 0 being best with no plaques and 3 having plaque covering more than half of the surface; white spots score: (0-3): 0 being no visible white spots and 3 having visible white spots requiring restoration.

^e^Percent over-BMI was calculated as [(BMI – BMI at 50th percentile for age and sex) / BMI at 50th percentile] × 100.

^f^UV: ultraviolet.

^g^HPV: human papillomavirus.

^h^Statistically significant *P*<.05.

#### Effects on Clinic Attendance

Narring et al found no significant differences in multidisciplinary clinic attendance as a result of text message reminders in comparison to control at 6-month follow-up across all clinics (d=0.15; 95% CI –0.03 to 0.33) or by clinic type; primary care (d=0.06; 95% CI –0.16 to 0.27), gynecology (d=0.32; 95% CI –0.10 to 0.74), or mental health clinics (d=0.32; 95% CI –0.21 to 0.85) [35]. In contrast, Branson et al reported a significant improvement in their mental health clinic attendance rate in the texting intervention group compared with controls at 3-month follow-up (d=0.67; 95% CI 0.09-1.25) [36].

#### Effects on Contraception

Castano et al found significantly higher oral contraceptive pill (OCP) continuation rates at 6 months (ie, having taken a pill in last 7 days) in texting intervention arm compared with controls (d=0.23; 95% CI 0.06-0.40) [37]. The observed effect of the intervention dissipated over time with the difference in OCP continuation rates significant at 187 days or less (d=0.52; 95% CI 0.19-0.86), but not at 188 days or more (d=0.13; 95% CI –0.07 to 0.33) [37]. In contrast, Hou et al reported no significant differences in average rates of missed OCPs in text intervention group compared with controls, overall (d=0.09; 95% CI –0.34 to 0.53) and in those who completed 3 cycles or 3-month follow-up (d=0.13; 95% CI –0.30 to 0.57) [38]. Similarly, Trent et al found that on-time Depo-Provera (injectable contraceptive) completion rate was not significantly different between text intervention and control groups over 12-month study period in those who completed cycle 1 (d=0.29; 95% CI –0.19 to 0.77), cycle 2 (d=0.77; 95% CI –0.35 to 0.69), or cycle 3 (d=0.01; 95% CI –0.57 to 0.60) [39].

#### Effects on Risky Behavior

Testing a texting intervention using a sequence of messages to assess and then intervene on risk behavior, Suffoletto et al reported no significant differences between intervention and control groups for condom use at last sexual intercourse (d=0.32; 95% CI –0.29 to 0.93) or proportion of “always condom use” over the past 28 days (d=0.29; 95% CI –0.38 to 0.95) at 3-month follow-up [40]. Additionally, there was no significant difference in observed drug or alcohol use before last sex (d=0.23; 95% CI –0.53 to 0.99) and any unprotected sex with concurrent alcohol use in last 28 days (d=0.58; 95% CI –0.41 to 1.57) [40]. Cornelius et al, in their evaluation of texting intervention among African American adolescents at 3-month follow-up, failed to show a significant improvement in human immunodeficiency virus (HIV) knowledge (d=0.42; 95% CI –0.03 to 0.86), attitudes toward condoms (d=0.08; 95% CI –0.36 to 0.52), and risky behavior related to sexual intercourse (d=0.17; 95% CI –0.27 to 0.60) or illegal drug use (d=0.41; 95% CI –0.03 to 0.86) [41]. In contrast, Lopez at al developed and evaluated a dedicated mobile phone app “DoctorChat” as a tool to provide sexual education [42]. At 6-month follow-up, the authors reported some improvement in participants’ knowledge about the sexual risk factors among young adults, but no significant differences in preventive behavior [42].

In an intervention using a single text message summarizing alcohol-related expenses by the participants over the prior month, Moore et al reported no significant reduction in average units of alcohol consumed at follow-up between intervention and control groups among students (d=0; 95% CI –0.65 to 0.65) and nonstudents participants (d=0.13; 95% CI –0.48 to 0.75) [43]. In contrast, Haug et al utilized a combined intervention of automatically generated individually Web-based feedback and text messages tailored for participants’ age, sex, and alcohol drinking behavior [44]. At 3-month follow-up, the authors were able to show a significant reduction in the number of risky single-occasion drinking episodes in persons with at least one occasion in the last month (d=0.22; 95% CI 0.01-0.42), but not the number of drinks or alcohol-related problems [44].

In another study, Haug et al evaluated the efficacy of a 2-way text message–based intervention on smoking cessation tailored based on individual smoking behavior and attitudes toward smoking cessation [45]. The authors showed a significant reduction in cigarette consumption (ie, the number of cigarettes smoked) at 6-month follow-up in all participants (d=0.33; 95% CI 0.16-0.50), occasional smokers (d=0.36, 95% CI 0.02-0.71), and daily smokers (d=0.20; 95% CI 0.01-0.39), as well as a significant increase in the number of smoking quit attempts in occasional smokers only (d=1.09; 95% CI 0.65-1.52) [45]. However, there was no significant improvement in either 7-day or 4-week smoking abstinence among study participants [45].

#### Effects on Oral Health

Zotti et al utilized an existing commonly used mobile phone app platform (WhatsApp) and showed a significant improvement in participants’ oral hygiene with lower average gingival index scores in the intervention group compared with controls at 6 months (d=0.56; 95% CI 0.12-1.01), 9 months (d=1.04; 95% CI 0.57-1.51), and 12 months (d=1.43; 95% CI 0.94-1.92) [46]. Intervention participants also had significantly lower plaque index scores at 6 months (d=0.73; 95% CI 0.28-1.18), 9 months (d=1.50; 95% CI 1.00-2.00), and 12 months (d=1.40; 95% CI 0.91-1.89) compared with controls [46]. In addition, the number of visible white spots was lower in the intervention group at 9 months (d=0.67; 95% CI 0.04-1.30) and 12 months (d=0.63; 95% CI 0.06-1.20) compared with controls [46]. However, the frequency of new caries was not significantly different between study groups [46]. Testing a less intensive texting intervention, Bowen et al reported significantly lower average plaque coverage score in the text intervention group compared with controls at 4-week (d=1.62; 95% CI 0.98-2.26); and 12-week follow-up (d=2.40; 95% CI 1.67-3.12) [47].

#### Effects on Weight Management and Physical Activity

Using a texting reminder approach, Lau et al reported a nonsignificant increase in the average self-report of physical activity scores in the intervention group compared with controls at 2-month follow-up (d=0.31; 95% CI –0.14 to 0.76) [48]. Abraham et al also failed to show any significant changes in body mass index (BMI) at 6-month follow-up in the 3 study groups exposed to a combination of Internet-based educational program and texting intervention versus controls (d=0.09; 95% CI –0.61 to 0.78) [49]. Similarly, testing a multifunction mobile phone app, Pretlow et al reported a nonsignificant improvement in self-management skills and weight loss (% over-BMI) at 5-month follow-up in the intervention group compared with controls, in neither males (d=0.17; 95% CI –0.55 to 0.88) nor females (d=0.08; 95% CI –0.44 to 0.61) [50]. In contrast, Direito et al, in their evaluation of the effect of 2 commercially available fitness apps—immersive app “Zombies, Run!” and nonimmersive app “Get Running-Coach” —on cardiopulmonary fitness among physically inactive adolescents [51] at 2 month follow up, failed to show a significant difference in the time needed to complete 1-mile run or walk using an immersive app (d=0.24; 95% CI –0.43 to 0.9) or nonimmersive app (d=0.14, 95% CI –0.5 to 0.81) compared with the control group.

#### Effects on Sun-Protective Measures

Sachse et al used a combined approach of a single educational session and texting intervention and reported, at 2-month follow-up, a significant increase in participants’ understanding of the meaning of UV-index (d=1.49; 95% CI 0.61-2.37), and their ability to name at least three of the ABCDE mnemonic (ie, asymmetry, border, color, diameter, evolution) for skin self-exam (d=1.40; 95% CI 0.19-2.61), but not in knowing that it takes hours to recognize sunburns (d=0.51; 95% CI –0.24 to 1.26) compared with their baseline [52].

#### Effects on Vaccination

Using a texting approach, Matheson et al reported significantly higher HPV vaccination series completion rate in intervention group compared with controls for HPV second dose (d=1.10; 95% CI 0.67-1.52), HPV second dose on time (d=0.46; 95% CI 0.06 to 0.86), HPV third dose (d=0.70; 95% CI 0.14-1.27), and HPV third dose on time (d=0.91; 95% CI 0.24-1.58) [53]. Similar significant beneficial effects were seen in the intervention group compared with those who were interested but not enrolled, regarding their completion rates of HPV second dose (d=0.93; 95% CI 0.40-1.46) and HPV third dose (d=1.24; 95% CI 0.05-2.42), but not HPV second dose on time (d=0.46; 95% CI –0.09 to 1.01) or HPV third dose on time (d=1.17; 95% CI –0.03 to 2.36) [53].

## Discussion

### Principal Findings

Prevention has been emphasized as a key component of adolescent health with evidence that many problem behaviors are amendable to intervention [4] Given the increasing use of communication technologies and mobile devices among young people [24], these media present opportunities for behavioral intervention. However, few studies have attempted to assess the efficacy of these approaches over different preventive behaviors. In this systematic review, we assessed the weight of evidence to date for 2 of the most common mobile technologies used by youth, texting and mobile phone apps, to promote preventive behaviors.

Overall, the evidence was modest, but limited with a small number of studies, relatively small sample sizes, and other methodological considerations, particularly for statistical analysis. We identified only 19 studies that met our pre-set criteria, the vast majority of which were texting interventions. These approaches were used to impact 7 types of behavior (clinic attendance, contraceptive use, risky behavior, oral health, physical activity and weight management, sun protection, HPV vaccination). Most interventions were carried out among younger adolescents and in clinic settings, which indicates the potential of clinical settings to adopt innovative technology-based prevention approaches to address different types of preventive behaviors.

Although texting was used more frequently than mobile phone apps to promote preventive behavior, there were many differences in the timing and content of texting approach. The majority of interventions used customized messages aimed at specific behavioral targets (depending on the preventive behavior of interest) and schedules. More than a simple reminder, texting was used to communicate educational messages, behavioral goals, and reinforce positive behavior. Many studies demonstrated both feasibility and satisfaction with these approaches, which suggests potential for further development.

Overall results for behavior change are modest, with half of interventions reviewed herein demonstrating evidence of efficacy. There was some evidence of efficacy for texting to promote oral contraceptive adherence, specialty clinic attendance, and HPV vaccination. Effects were strongest for oral health and hygiene with both a multifunction app and a texting approach, resulting in significant effects. There was limited evidence of efficacy of either a multifunction app or texting approach on weight management and physical activity; or for texting approach to change sexual risk behavior in the context of substance use. The variability in the observed effects across different behaviors could be due to the level of difficulty and effort required. The challenges and the characteristics associated with certain behaviors would make them easier or harder to influence or change over time. More research is needed to measure the level of difficulty of behavior change in a standardized way and to compare effect sizes across behaviors and intervention approaches.

A recent review of electronic media to promote health or safety behavior change in children (aged ≤18 years) concluded that there is good evidence of efficacy for these approaches [54]. Most studies focused on pre-teen children and utilized computer games and videos. The difference in findings in comparison to our review may be due in part to our focus on adolescents, for whom behaviors may be more difficult to change; our focus on newer technologies, which are still relatively nascent media for health behavior change; and on a wide variety of preventive behaviors, for which there is heterogeneity in approach. Mobile phone approaches, including texting in particular, have been found to be a feasible and potential efficacious medium for increasing levels of health education in adolescents [55]; behavioral targets may be more resistant to change.

### Strengths

Our systematic review had a number of strengths. First, in our review, we followed the recommendations for rigorous systematic reviews methodology [28,29,56-58]. Second, we conducted a review with a highly sensitive search strategy guided by a librarian information specialist with no language restrictions to minimize publication bias and identify the largest possible number of relevant studies. Our search also included published systematic reviews, clinical trial registries, and various electronic databases. Third, although our search was limited to studies published since 1995, we identified no eligible studies before 2005, and therefore we believe that the possibility of missing earlier studies is very small. Finally, 2 authors completed the review process independently at all stages of the systematic review.

### Limitations

Some potential methodological limitations of our systematic review warrant discussion. First, similar to any other systematic literature review, although our search criteria were planned to be as comprehensive as possible, the possibility of missing few relevant articles cannot be excluded. Second, to identify the strongest available evidence, we included only articles that were published in peer-reviewed journals, and therefore there could be a publication bias with the tendency to report positive study results [59]. Third, the study sample size and ages, and the definition of adherence to preventive behaviors and other related outcomes varied. These limitations prohibited a meta-analysis from being performed [60]. Fourth, a number of the included studies had relatively small sample sizes and short follow-up period. Finally, the number of the studies that met our predefined criteria was relatively small; however, this is likely the result of the available evidence and published studies in the field.

### Conclusions

In conclusion, despite the promising feasibility and acceptability data of texting and mobile phone apps in improving preventive behavior among adolescents, the evidence for actual behavior change is modest, with most studies of relatively low to moderate quality. However, the field of mobile health research is an evolving one with promising results that suggest a potential impact on improving health outcomes, given the growing evidence and the ubiquitous access to mobile technology. The variability in the observed effects across studies could be related to the nature of different behaviors and the heterogeneity of intervention approaches. Further research of these intervention approaches with rigorous research designs is needed to evaluate their efficacy, effectiveness, and cost-effectiveness in promoting preventive behavior among adolescents. These research efforts would be crucial to inform the evidence base on the use of texting and mobile phone apps as tools for behavior change among adolescents.

## Appendix

Multimedia Appendix 1 Updated search strategies.

Multimedia Appendix 2 Eligibility criteria of the included studies.

Multimedia Appendix 3 Detailed description of text message interventions.

Multimedia Appendix 4 Detailed description of mobile phone app interventions.

Multimedia Appendix 5 Outcomes of text message interventions.

Multimedia Appendix 6 Outcomes of mobile phone app interventions.

## Abbreviations

BMI: Body Mass Index
ED: Emergency Department
HIV: Human Immunodeficiency Virus
HPV: Human Papillomavirus
OCP: Oral Contraceptive Pills
PRISMA: Preferred Reporting Items of Systematic Reviews and Meta-analyses
RCT: Randomized Controlled Trial
RSOD: Risky Single-occasion Drinking
SD: Standard Deviation
SMD: Standardized Mean Differences
SMS: Short Message Service
STI: Sexually Transmitted Infection
UV: Ultraviolet
