# Supplementary material for: Texting and Mobile Phone App Interventions for Improving Adherence to Preventive Behavior in Adolescents: A Systematic Review
Source: JMIR Mhealth Uhealth. 2017 Apr 19;5(4):e50. doi: 10.2196/mhealth.6837 (PMC5415660; doi:10.2196/mhealth.6837)
Supplement: Multimedia Appendix 1 [file mhealth_v5i4e50_app1.pdf]

PubMed – searched 7.29.15

(((("text messaging"[mesh] OR "cell phones"[mesh] OR app[All Fields] OR apps[All Fields] OR "mobile phone*"[All Fields] OR "cell phone*"[All Fields] OR text*[All Fields]))

AND (adherence[All Fields] OR compliance[All Fields]))

AND ("Young Adult"[Mesh] OR "Adolescent"[Mesh] OR "Child"[Mesh] OR pediatric*[All Fields] OR child*[All Fields] OR adolescen*[All Fields] OR youth*[All Fields])

Results: 253

Embase – searched 8.5.2015

(‘text messaging’/exp OR ‘texting’ OR 'mobile phone'/exp OR ‘cell phone’ OR ‘cell phones” OR ‘cellular phone’ OR ‘cellular telephone’ OR ‘mobile telephone’ OR 'mobile application'/exp OR ‘mobile app’ OR ‘mobile applications’ OR ‘mobile apps’ OR ‘portable software app’ OR ‘portable software application’ OR ‘portable software applications’ OR ‘portable software apps’)

AND

('patient compliance'/exp OR ‘adherence’ OR ‘compliance’)

AND

('child'/exp OR child* OR 'juvenile'/exp OR adolescen* OR teenage* OR youth* OR pediatric*)

Results: 313

Cochrane CENTRAL – searched 8.5.2015

Add to top | View fewer lines

#	Search	Results
#1	MeSH descriptor: [Text Messaging] explode all trees	160
#2	MeSH descriptor: [Cell Phones] explode all trees	463
#3	app or apps or "mobile phone*" or "cell phone*" or text*	17419
#4	adherence or compliance	34155
#5	MeSH descriptor: [Young Adult] explode all trees	278
#6	MeSH descriptor: [Adolescent] explode all trees	77687
#7	MeSH descriptor: [Child] explode all trees	188
#8	pediatric* or child* or adolescen* or youth*	173003
#9	#1 or #2 or #3	17419
#10	#5 or #6 or #7 or #8	173116
#11	#9 and #4 and #10	2316
#12		N/A

Cochrane CENTRAL: 137

CINAHL – Searched 8.5.2015

#	Query	Limiters/Expanders	Last Run Via	Results
S10	S7 AND S8 AND S9	Search modes - Boolean/Phrase	Interface - EBSCOhost Research Databases Search Screen - Basic Search Database - CINAHL with Full Text	101
S9	S5 OR S6	Search modes - Boolean/Phrase	Interface - EBSCOhost Research Databases Search Screen - Basic Search	514,746

			Database - CINAHL with Full Text	
S8	S3 OR S4	Search modes - Boolean/Phrase	Interface - EBSCOhost Research Databases<br>Search Screen - Basic Search<br>Database - CINAHL with Full Text	53,397
S7	S1 OR S2	Search modes - Boolean/Phrase	Interface - EBSCOhost Research Databases<br>Search Screen - Basic Search<br>Database - CINAHL with Full Text	16,764
S6	child* OR adolescen* OR teenage* OR youth* OR pediatric*	Search modes - Boolean/Phrase	Interface - EBSCOhost Research Databases<br>Search Screen - Basic Search<br>Database - CINAHL with Full Text	493,231
S5	(MH "Young Adult") OR (MH "Adolescence") OR (MH "Child")	Search modes - Boolean/Phrase	Interface - EBSCOhost Research Databases<br>Search Screen - Basic Search<br>Database - CINAHL with Full Text	365,205
S4	adherence OR compliance	Search modes - Boolean/Phrase	Interface - EBSCOhost Research Databases	53,397

			Search Screen - Basic Search Database - CINAHL with Full Text	
S3	(MH "Medication Compliance") OR (MH "Patient Compliance")	Search modes - Boolean/Phrase	Interface - EBSCOhost Research Databases Search Screen - Basic Search Database - CINAHL with Full Text	24,430
S2	app OR apps OR mobile phone* OR cell phone* OR text*	Search modes - Boolean/Phrase	Interface - EBSCOhost Research Databases Search Screen - Basic Search Database - CINAHL with Full Text	16,469
S1	(MH "Text Messaging") OR (MH "Cellular Phone") OR (MH "Mobile Applications") OR (MH "Smartphone")	Search modes - Boolean/Phrase	Interface - EBSCOhost Research Databases Search Screen - Basic Search Database - CINAHL with Full Text	

CINAHL: 101

PsycINFO – searched August 11, 2015

S4	S1 AND S2 AND S3	Search modes - Boolean/Phrase	Interface - EBSCOhost Research Databases	153

			Search Screen - Basic Search Database - PsycINFO	
S3	(child* OR juvenile OR adolescen* OR teenage* OR youth* OR pediatric*)	Search modes - Boolean/Phrase	Interface - EBSCOhost Research Databases Search Screen - Basic Search Database - PsycINFO	827,019
S2	DE "Compliance" OR compliance OR adherence	Search modes - Boolean/Phrase	Interface - EBSCOhost Research Databases Search Screen - Basic Search Database - PsycINFO	40,700
S1	(DE "Mobile Devices") OR (DE "Cellular Phones") OR "text messaging" OR "cell phone*" OR app OR apps OR "mobile phone*" OR text*	Search modes - Boolean/Phrase	Interface - EBSCOhost Research Databases Search Screen - Basic Search Database - PsycINFO	101,572

PsycINFO: 153

Totals as of August 11, 2015

PubMed: 432
Embase: 313
CINAHL: 101
Cochrane Central: 137
PsycINFO: 153

Total: 1136

Deduped against PubMed: removed 373

Unique remaining (non-PubMed): 331

Web of Science: searched Sep 17, 2015

((text messaging OR texting OR mobile phone OR cell phone OR cell phones OR cellular phone OR cellular telephone OR mobile telephone OR mobile application OR mobile app OR mobile applications OR mobile apps OR portable software app OR portable software application OR portable software applications OR portable software apps)) *AND* **TOPIC:** ((“patient compliance” OR “adherence” OR “compliance”)) *AND* **TOPIC:** ((“child” OR child* OR “juvenile” OR adolescen* OR teenage* OR youth* OR pediatric*))

Results: 169

CRD: searched Sep 17, 2015

(text messaging OR texting OR mobile phone OR cell phone OR cell phones OR cellular phone OR cellular telephone OR mobile telephone OR mobile application OR mobile app OR mobile applications OR mobile apps OR portable software app OR portable software application OR portable software applications OR portable software apps)

AND

(patient compliance OR adherence OR compliance)

AND

(child OR child* OR juvenile OR adolescen* OR teenage* OR youth* OR pediatric*)

Results: 10 (all from DARE) – also included search of NHTA database – no results

---

Inspec: searched Sep 17, 2015

(text messaging OR texting OR mobile phone OR cell phone OR cell phones OR cellular phone OR cellular telephone OR mobile telephone OR mobile application OR mobile app OR mobile applications OR mobile apps OR portable software app OR portable software application OR portable software applications OR portable software apps)

AND

(patient compliance OR adherence OR compliance)

AND

(child OR child* OR juvenile OR adolescen* OR teenage* OR youth* OR pediatric*)

INSPEC: 23

IEEE: November 6, 2015

Simplified search because IEEE will not support more than 15 search terms.

(“text messaging” OR mobile OR “cell phone” OR “cell phones” OR portable)

AND

(adherence OR compliance)

AND

(child OR child* OR juvenile OR adolescen* OR teenage* OR youth* OR pediatric*)

IEEE: 16

Grey Literature search

Proquest Dissertations: November 6, 2015

(“text messaging” OR texting OR “mobile phone” OR “mobile phones” OR “cell phone” OR “cell phones” OR “cellular phone” OR “cellular telephone” OR “mobile telephone” OR “mobile application” OR “mobile app” OR “mobile applications” OR “mobile apps” OR “portable software app” OR “portable software application” OR “portable software applications” OR “portable software apps”)

AND

(adherence OR compliance)

AND

(child OR child* OR juvenile OR adolescen* OR teenage* OR youth* OR pediatric*)

Proquest: 6 results

Scopus: November 6, 2015

(“text messaging” OR texting OR “mobile phone” OR “mobile phones” OR “cell phone” OR “cell phones” OR “cellular phone” OR “cellular telephone” OR “mobile telephone” OR “mobile application” OR “mobile app” OR “mobile applications” OR “mobile apps” OR “portable software app” OR “portable software application” OR “portable software applications” OR “portable software apps”)

AND

(adherence OR compliance)

AND

(child OR child* OR juvenile OR adolescen* OR teenage* OR youth* OR pediatric*)

Scopus: 663

ClinicalTrials.gov: searched November 6, 2015

(text messaging OR mobile OR cell phone OR cell phones)

AND

(adherence OR compliance)

AND

(child OR child* OR juvenile OR adolescen* OR teenage* OR youth* OR pediatric*)

ClinicalTrials.gov: 237 documents

Controlled Clinical Trials: searched November 6, 2015 – 2 results

---

WHO Clinical Trials: searched November 6, 2015

(text messaging OR mobile OR cell phone OR cell phones)

AND

(adherence OR compliance)

0 results when add child; 5 results when leave out. Went with 5 results.

Google Scholar: searched November 21, 2015

Suggested search strategies:

allintitle: text messaging adherence child OR children OR juvenile OR adolescent OR teenager OR youth OR pediatric (n=5)

allintitle: mobile adherence child OR children OR juvenile OR adolescent OR teenager OR youth OR pediatric (n=8)

allintitle: SMS adherence child OR children OR juvenile OR adolescent OR teenager OR youth OR pediatric (n=1)

allintitle: portable adherence child OR children OR juvenile OR adolescent OR teenager OR youth OR pediatric (n=2)

Google scholar: 15 results

Journal of Medical Internet Research (JMIR): searched February 2, 2017

Theme 188: E-collection "mHealth for Wellness, Behavior Change and Prevention"

Link: https://mhealth.jmir.org/themes/188

Theme 189: E-collection "Text-messaging (SMS)-Based Interventions"

Link: https://mhealth.jmir.org/themes/189

Total number of articles identified in JMIR that included adolescents: 31
