# Supplementary material for: Texting and Mobile Phone App Interventions for Improving Adherence to Preventive Behavior in Adolescents: A Systematic Review
Source: JMIR Mhealth Uhealth. 2017 Apr 19;5(4):e50. doi: 10.2196/mhealth.6837 (PMC5415660; doi:10.2196/mhealth.6837)
Supplement: Multimedia Appendix 2 [file mhealth_v5i4e50_app2.pdf]

Supplemental - Table 1. Eligibility criteria of the included studies

Source (Country)	Selection Criteria
Narring et al. [35] (Switzerland)	• Age 12-24 years<br>• All adolescent patients registered for an appointment in the youth clinic<br>• Have their phone numbers recorded in clinic<br>• Patients could opt out of study
Branson et al. [36] (United States)	• Age 13-17 years<br>• Attended outpatient individual and/or group therapy<br>Intervention:<br>• Owned a cellular phone with text-messaging capabilities<br>• Newly admitted or existing patients<br>Controls:<br>• Consecutive admissions to same clinic beginning on the first day of enrollment in the intervention arm
Castano et al. [37] (United States)	• Age < 24 years<br>• Sexually active<br>• Owned cell phone with text messaging functionality<br>• No medical contraindication for oral contraceptive pills
Hou et al. [38] (United States)	• Sexually active<br>• New users of oral contraceptive pills for contraception (<1month start date)<br>• Plan to use oral contraceptive pills for at least 6 months<br>• Have a personal mobile phone with text messaging capabilities<br>• No plans to travel outside United States/Canada during study period
Trent et al. [39] (United States)	• Age 13-21 years<br>• Willing to be randomized<br>• Currently using Depo-Provera injections<br>• Have a personal cell phone with text messaging capability
Suffoletto et al. [40] (United States)	• Age 18-25 years<br>• Self-report of hazardous drinking behavior<br>• Report of 1 or more of the following:<br>o >1 male sex partner in past 3 months<br>o No condom use at last sexual intercourse<br>o Alcohol/drug use concurrent with their last sexual intercourse<br>• No substance abuse<br>• No psychiatric problems<br>• No pregnancy plans in next 3 month<br>• Have personal mobile phone with text messaging functionality
Cornelius et al. [41] (United States)	• African American adolescents<br>• Age 13-18 years of age<br>• Provided verbal and written assent or consent<br>• Had parental consent to participate<br>• Had knowledge of mobile cell phone text messaging technology
Lopez et al. [42] (Colombia)	• Age 18-29 years<br>• Reside in Colombia.<br>• Have access to a mobile device that:<br>o Allows navigation using a wireless network or an owned data plan<br>o Allows the installation of the app (installation requirements were provided)<br>• Have read, understood, and accepted the terms and conditions of the study

Moore et al. [43] (Wales)	• Students and non-students university members<br>• Have a valid e-mail address<br>• Participated in previous university research study<br>• Expressed earlier interest in future research
Haug et al. [44] (Switzerland)	• Ownership of a mobile phone
Haug et al. [45] (Switzerland)	• Daily or occasional cigarette smoking (at least 4 cigarettes in the preceding month and at least 1 cigarette during the preceding week)<br>• Ownership of a mobile phone<br>• Students in vocational schools were proactively invited to participate, irrespective of their intention to quit
Zotti et al. [46] (Italy)	• Started orthodontic multibracket treatment<br>• Own a smartphone<br>• Were able to be online daily<br>• No restrictive dietary regimen<br>• No other existing medical conditions
Bowen et al. [47] (United States)	• Age 10-18 years<br>• Had access to a cellular phone<br>• Received orthodontic treatment with fixed maxillary edgewise appliances<br>• Had at least 6 months of remaining orthodontic treatment
Lau et al. [48] (Hong Kong)	• Age 12-15 years<br>• Internet access at home<br>• Had a personal mobile phone<br>• No physical or psychological limitation that would preclude physical activity<br>• Not meeting physical activity recommendations at the time of enrollment
Abraham et al. [49] (Hong Kong)	• Age 12-18 years<br>• Having a BMI greater than 95th percentile for age<br>• Attending the Paediatric Obesity and Lipid Clinic<br>• Being proficient in Cantonese
Pretlow et al. [50] (United States)	• High motivation with a score of 50 or more in a 0-100 scale<br>• Willingness to attend group or phone meetings or weigh their foods at meals
Direito et al. [51] (New Zealand)	• Age 14-17 years<br>• Lived in Auckland, New Zealand<br>• Owned an iPod touch or smartphone running at least Android 2.2 or iOS 6.0<br>• Were able to perform physical activities but were not achieving the PA recommendations, e.g. at least 60 minutes of moderate-to-vigorous physical activity each day
Sachse et al. [52] (Germany)	• Participants in 2 summer camps (1-week each) for organ transplant recipients<br>• Had access to a mobile phone with text messaging functionality<br>• Demonstrated ability to retrieve text messages
Matheson et al. [53] (United States)	• Age 11-22 y<br>• Initiated human papilloma virus vaccination series during clinic visit and at the time of enrollment
