# Supplementary material for: Texting and Mobile Phone App Interventions for Improving Adherence to Preventive Behavior in Adolescents: A Systematic Review
Source: JMIR Mhealth Uhealth. 2017 Apr 19;5(4):e50. doi: 10.2196/mhealth.6837 (PMC5415660; doi:10.2196/mhealth.6837)
Supplement: Multimedia Appendix 3 [file mhealth_v5i4e50_app3.pdf]

Supplemental - Table 2. Detailed description of text message interventions

Source (Country)	Detailed Intervention Description
Narring et al. [35] (Switzerland)	**Purpose:** Improve attendance in a multi-disciplinary clinic<br><br>**Intervention:**<br>• Generic text messages in French to all participants<br>• Sent between 8 and 11 am the day before scheduled appointment<br>• Customized for date/time of appointment and name of the doctor<br>• Two-way communication, patients could respond by "No" if they did not plan to attend their scheduled appointment<br>• Text messages automatically, based on scheduled date, through "Easy Smart Care" using EasyMed Services Inc. platform<br>• Research assistant and EasyMed Inc. managed text messages<br>• Was not possible to know if text messages were received<br>• No incentives or rewards for participation
Branson et al. [36] (United States)	**Purpose:** Improve attendance in mental health clinic<br><br>**Intervention:**<br>• Generic text messages to all participants<br>• Sent the night before scheduled therapy session<br>• Research staff used a study cell phone to send reminders<br>• Confidentiality maintained, no reference to nature of the appointment (e.g., "C u Wed @8")<br>• One-way communication, from research staff to patients<br>• Participant phone number was verified at time of enrollment by texting study cell phone one time<br>• Participants' phone numbers saved on study cell phone and deleted after completion of the study<br>• Participants offered $20 prepaid cellular phone minutes per month to offset cost of text reminders<br>• Participants received $20 gift-card after 3-mon follow up interview regarding satisfaction with intervention
Castano et al. [37] (United States)	**Purpose:** Improve continuation of oral contraceptive pills at 6 months<br><br>**Intervention:**<br>• Daily text messages for 180 days (6 months)<br>• Each participant decided her text-message start date and time of delivery in a given day<br>• Text messages sent through secure web-based platform<br>• Message content varied and included:<br>o Introductory message (n=1)<br>o How to change contact information or message time (n=3)<br>o Educational messages (n=47), repeated 4 times<br>o Two-way communications for quality control (n=12)<br>o Final message (n=1)<br>• Total individual message length under 160 characters<br>• Confidentiality maintained, no names or mention of oral contraceptive pills<br>• All text-messages were available in English and Spanish<br>• Participants could login to study web portal to change time of daily text messages, contact information, or discontinue text messages at any time<br>• Participants instructed to direct all medical questions to health care providers<br>• Participants received $10 compensation that cover the cost of text messages for 6 month study period<br><br>**Intervention and Control:**

	• SOC included counseling by staff, and educational handout that covered use, effectiveness, risks or side effects, benefits, and mechanism of action
Hou et al. [38]<br>(United States)	**Purpose:** Increase oral contraceptive pills adherence<br><br>**Intervention:**<br>• Daily text messages for 90 days (3 months)<br>• Generic message content "Please remember to take your birth control pill"<br>• Customized text delivery time based on participant preference<br>• Text sent through TXT Signal, Inc., Gainesville, FL<br><br>**Intervention and Control:**<br>• Received EMD (SIMPill) for real-time pill adherence assessment (Clinical Technology Advisors, Inc., Acton, MA)<br>• Were asked to charge their EMD once a month, with a reminder sent to them the 15th day of every month<br>• Completed paper pill diary for 3 month, returned at end of study<br>• Were allowed to use a phone or clock alarm as reminders<br>• Participants were allowed to chose from 2 oral contraceptive pills regimens
Trent et al. [39]<br>(United States)	**Purpose:** Improving Depo-Provera appointment attendance<br><br>**Intervention (DepoText):**<br>• Messages included:<br>o Welcome message<br>o Daily appointment reminder text starting 72 hours before appointment date<br>o Education on condom use for STI prevention, weight management, encouragement to call with problems, and a reminder for STI screening<br>• Generic message content to all participants with nurse case manager signature at the end of the text<br>• Message delivery and patient responsiveness were tracked<br>• Two-way communication as patients responded "Yes" or "No" if they plan to attend appointment, if "No", e-mail sent to nurse to reschedule<br>• $10 remuneration for completing baseline web-based survey, and $5 if they notified nurse manager with any change in their contact information<br>• Confidentiality was relatively maintained, no patient names, but content was related to Depo-Provera use and sexual health<br>• Depo-Text sent through Compliance for Life short messaging system platform (iReminder, LLC, Westfield, NJ)<br><br>**Intervention and Control:**<br>• SOC included nursing assessment, medical assessment if indicated, counseling, appointment card with next injection date, and automated clinic appointment reminders to their home phones
Suffoletto et al. [40]<br>(United States)	**Purpose:** Reduce sex risk behavior among at-risk young adult females discharged from emergency department<br><br>**Intervention:**<br>• Participants received initial welcome messages describing the program at time of enrollment<br>• Each Sunday at noon, a sequence of text messages was sent to:<br>o Assess risky encounters over the past week<br>o Provide personalized feedback on risk behavior<br>o Prompt collaborative goal setting to not have a risky encounters over the coming week<br>• Text messaging algorithm:<br>o If no response to weekly queries within 6 hours, a second text was sent with the same initial assessment message

	o If no response in 12 hours following the second message, data were considered lost and participants were re-texted the following week<br>o If no assessments completed for 2 weeks, participants were e-mailed to check their contact information (e.g. phone number)<br>• Informative messages sent in-between weekly assessments to increase individual's perceived:<br>o Susceptibility of getting STIs<br>o Severity of health risk associated with STIs<br>o Benefits of adopting protective behaviors (e.g. using condoms)<br>• Feasibility based on eligibility, enrollment, and completion rates<br>• Intervention group evaluated acceptability based on a survey of the value of provided information and intervention usefulness<br><br>**Control:**<br>• Received a welcome text message, and follow up messages with how many weeks left in the study<br><br>**Intervention and control:**<br>• Logged into study website to complete follow-up final questionnaire that measured alcohol use and sex behaviors, similar baseline questionnaire<br>• Participants received $10 after completing baseline questionnaire, and $20 after completing final questionnaire
Cornelius et al. [41] (United States)	**Purpose:** Improve HIV knowledge and attitudes toward condoms among African American adolescents<br><br>**Intervention:**<br>• Becoming a Responsible Teen (BART) curriculum<br>o Seven weekly sessions conducted at 10 AM on Saturdays at the University<br>o Information sessions covered the topics of understanding HIV, sexual decision making, developing condom skills, learning and practicing assertiveness communication skills, personalizing risks, and spreading the word about BART<br>o Each session lasted for about 90 to 120 minutes<br>o Final session (graduation) at the 3-month follow-up and participants took post-BART surveys on the computer<br>• Text messaging:<br>o Started the day after completion of BART curriculum<br>o Daily multi-media mobile cell phone boosters (text messages, pictures, and videos)<br>o All messages were delivered at 3 PM daily for 3 months<br>o Participants were required to respond to each message and the assigned facilitator sent tailored responses to each reply<br>o Participants were also allowed to text their assigned facilitators with additional questions about the process or any topic related to safer sex practices<br>o Each participant were provided a smart phone with unlimited text messaging and Web access for 90 days<br>o At the end of 3 months of texting, participants returned for a follow-up session, completed follow- up surveys on the computer, and attended a graduation ceremony (session 8 of the BART curriculum)<br>• Facilitators:<br>o Two trained master facilitators were responsible for training other facilitators and sending daily text messages to participants<br>o Six additional facilitators were hired to deliver the face-to-face information and to respond to participants' text messaging responses<br>o Required to be African American, have some college education or experience working with adolescents, and have effective communication skills<br>o Trained to deliver the BART curriculum and to respond to participants' text message responses via smart phones

	• Participants were compensated $20 for each weekly session and $50 for the 3-month follow-up session
Moore et al. [43] (Wales)	**Purpose:** Reduce future alcohol consumption based on data of past alcohol expenditure<br><br>**Intervention:**<br>• Definition of different alcohol units was sent to participants<br>• Daily text messages sent at 11 am requesting alcohol consumption data, in units, in the preceding day<br>• Participants could leave the study and stop receiving text messages any time<br>• Intervention was a single text message intervention sent to participants half-way through the study (week-4) with their alcohol expenditure in the previous month, calculated based on their reported consumption and average price unit<br>• Intervention message read "Alcohol study: We estimate that you have spent £x on alcohol in the past month"
Haug et al. [44] (Switzerland)	**Purpose:** Reduce alcohol binge or problem drinking in vocational school students.<br><br>**Intervention:**<br>• Alk-Check, automatically generated individually tailored online feedback and SMS messages<br>• Content of both components was based on effective social norms intervention programs<br>• Online program:<br>o Tailored according to: gender, age, number of standard drinks in a typical week, and frequency of risky single-occasion drinking (RSOD) occasions in the last 30 days.<br>o Feedback included graphical and textual information concerning (1) drinks per week in relation to the age and gender-specific reference group, (2) financial costs of drinking, (3) calories consumed with alcoholic drinks, and (4) number of heavy drinking occasions in relation to the age and gender-specific reference group<br>o Included an option to print and send electronically to the participants' email accounts<br>• Text messages:<br>o All participants received text messages for a period of 12 weeks<br>o Participants were assigned to one of three risk groups (Non-risk, Low-Risk or High-Risk), based on the number of RSOD occasions in the last 30 days<br>o Messages were sent either weekly (low risk) or weekly + bi-weekly (high risk)<br>o Content and number of text messages were tailored according to: gender, motivation for reduced alcohol consumption, alcohol-related problems, typical drinking day and time, number of standard drinks in a typical week, and maximum number of drinks on a single occasion during the last 30 days<br>• Program participants would take part in a draw for 10 vouchers worth €50
Haug et al. [45] (Switzerland)	**Purpose:** Increase smoking cessation and reduce cigarettes consumption in vocational school students<br><br>**Intervention:**<br>• SMS-COACH, an SMS two-way text message-based intervention over a period of 3 months<br>• The intervention program consisted of:<br>1. An online assessment of individual smoking behavior and attitudes toward smoking cessation<br>2. A weekly SMS text message assessment of smoking-related target behaviors sent at a fixed time point each week (6 pm on the weekday of study registration). Participants replied with a single letter or number.<br>3. 2 weekly text messages tailored to the data of the online and the SMS text message assessments<br>4. An integrated quit day preparation and relapse-prevention program<br>• Included cognitive behavioral and motivational components that were based on the Health Action Process Approach (HAPA) and its different stages: 3 non-active stages (pre-contemplation, contemplation, and preparation) and 2 active stages (action and maintenance).

	• The HAPA stage was assessed in even weeks and the number of cigarettes smoked per day or week was assessed in odd weeks<br>• Content of text messages was tailored according to participants' HAPA stage and data gathered at baseline and a weekly assessments<br>• Participants received:<br>o A total of 37 text messages (1 welcome message, 11 assessment messages, 24 tailored feedback messages, 1 goodbye message).<br>o Plus additional 42 text messages if they used the quit day preparation and relapse-prevention as 2 daily text messages from 1 week before the scheduled quit date until 3 weeks afterwards<br>• All incoming and outgoing text messages were automatically recorded, and all incoming messages were analyzed immediately<br>• Equivalent of €8 was offered as reimbursement with an equivalent of €0.80 offered as reimbursement for each SMS text message response to the weekly SMS text message assessments in the program<br><br>**Control:**<br>• Participants did not receive any of the intervention elements of the SMS-COACH program<br>• Equivalent of €8 was offered as reimbursement
Bowen et al. [47]<br>(United States)	**Purpose:** Improve oral hygiene compliance and reduce plaque formation in orthodontic patients<br><br>**Intervention and Control:**<br>• Both watched an audiovisual presentation on how to properly brush with a conventional toothbrush<br><br>**Intervention:**<br>• Generic text messages 2-3 weekly for 4 weeks with a total of 12 messages, then once weekly for 8 weeks<br>• Messages emphasized the importance of oral hygiene and served as a reminder for brushing their teeth as well<br>• Text limit was 160 for all messages<br>• Automated text messages were sent through Televox platform<br>• No customization for time of delivery<br>• Participants names were not revealed in the text messages<br>• All teeth photographs both groups were taken with the same professional camera with digital image analysis by Digimizer
Lau et al. [48]<br>(Hong Kong)	**Purpose:** Promote physical activity in school age children<br><br>**Intervention:**<br>• Internet PA program accessed twice weekly:<br>o Utilized an existing previously tested program, Teenstep.com<br>• Text-messages:<br>o Daily text messages in weekdays<br>o Adapted from previous SMS-based health behavior interventions<br>o Constructed to imitate a colloquial dialogue with a virtual friend "Jackie" to establish rapport as human-human interactions<br>o Message topics focused on motivation, information, behavioral skills, re-enforcement of PA benefits or solutions for PA barriers<br>o Participants were offered monthly incentives (e.g. water bottles), and $30 every month to cover the costs of SMS messages<br>• School teachers facilitated the process of student enrollment and study assessments at baseline and at the end of the study
Abraham et al. [49]<br>(Hong Kong)	**Purpose:** Improve weight management

	**Text/Internet Intervention:**<br>• Usual care and web- or internet-based intervention<br>• 12-week online curriculum<br>• Quiz at the end of each lesson<br>• Cell phone text messages follow up over 6 months<br>• Participants set specific goals related to diet and physical activity (baseline and monthly)<br>• Weekly semi-personalized messages that incorporated participant's diet and physical activity goals<br>• Participants replied with an emotion icon to represent their status with their goals for that week<br><br>**Simplified Lifestyle Modification Intervention:**<br>• Usual care and four meetings with a nutritionist over three months<br>• Patient-centered approach and cognitive behavioral concepts to improve diet and exercise knowledge and ways to change long term lifestyle
Sachse et al. [52] (Germany)	**Purpose:** Improve sun-protection knowledge and behavior in organ transplant recipients<br><br>**Intervention:**<br>• In-person training session by a dermatologist:<br>o 5-hour dermatological sun protection training<br>o Theory-based topics were covered, including skin types according to photo-typing scale, UV-rays, UV-index, sunscreen use, textile photo-protection, skin self-examination, and applying mnemonic ABCDE when evaluating skin lesions<br>o UV-index was defined as the maximum daily level of local UV-rays<br>o UV-index in the daily text messages was called "sun protection traffic light", as green, yellow and red<br>o All participants examined for any skin or mucosal disorders<br>• Text messages:<br>o Sun protection recommendations were sent daily for 4 weeks<br>o Content of text reminders were derived from participants local UV-index, based on German Meteorological Service<br>o Each message had 2 components:<br>**1.** Individual UV-index traffic light and 10 am local weather forecast every day<br>**2.** Behavioral "prompt" or message, such as sun-safe clothing and wearing sunglasses
Matheson et al. [53] (United States)	**Purpose:** Increase HPV vaccination series completion rate<br><br>**Planning and enrollment:**<br>• Educational sessions with clerical, clinical and provider groups in clinic to explain the goals and flow of the project<br>• Clinical staff provided patients/parents with HPV vaccination information sheets, and providers discussed it afterwards<br>• If patients/parents initiated the HPV vaccine, text messages enrollment was offered<br>• Participants completed enrollment form, but also had to complete an opt-in process using their personal phones<br>• Patients were enrolled in the study over a period of 3 month, with a total follow up of 8 month<br><br>**Intervention:**<br>• Three generic text messages were sent per HPV vaccine dose: 7 days before due date, on due date, and 7 days after due date<br>• Message was always the same and read "You are due for your next vaccine dose. Please call our office at xxx-xxx-xxxx if you have not yet completed a vaccine only appointment"<br>• Automated messages were sent through Call-Em-All platform

Note: Control groups were included in the table only if they had important relevant details to describe.

EMD: electronic monitoring device
HPV: human papilloma virus
PA: physical activity
STIs: sexually transmitted infections
UV: ultra-violet
