# Supplementary material for: Texting and Mobile Phone App Interventions for Improving Adherence to Preventive Behavior in Adolescents: A Systematic Review
Source: JMIR Mhealth Uhealth. 2017 Apr 19;5(4):e50. doi: 10.2196/mhealth.6837 (PMC5415660; doi:10.2196/mhealth.6837)
Supplement: Multimedia Appendix 4 [file mhealth_v5i4e50_app4.pdf]

Supplemental - Table 3. Detailed description of mobile phone app interventions

Source (Country)	Detailed Intervention Description
Lopez et al. [42] (Colombia)	**Purpose:** Promote and deliver accurate sexual health information that would provide sexual education and improve knowledge about the sexual risk factors among young adults<br><br>**Intervention:**<br>• DoctorChat is an app that allowed users to send inquiries on sexual and reproductive health topics through their mobile phones, and to receive personalized and accurate responses from a knowledgeable group of physicians<br>• The app ran on 4 mobile platforms (iOS/iPhone, Android, RIM/Blackberry, and Symbian)<br>• Once registered, participants received an automatic confirmation via email with their personal username and password to access the online survey<br>• After completion of the pre-intervention online questionnaire, participants were able to download and use the DoctorChat mobile app<br>• After the 6-month period, participants completed the post-intervention survey, which included questions on satisfaction with the app, to conclude the process
Zotti et al. [46] (Italy)	**Purpose**: Improve oral hygiene compliance and oral health in orthodontic patients<br><br>**Intervention and Control:**<br>• Both received standard of care, including:<br>o Standardized oral hygiene instructions<br>o Oral hygiene kit (toothpaste, toothbrush, mouthwash, interproximal brush, dental floss, and plaque-disclosing tablets)<br><br>**Intervention**:<br>• Had WhatsApp-based anonymous chat room "Brush Game"<br>• Chat room was moderated by one of the study investigators<br>• Patients used fictional names, and were forbidden from sharing photographs or information that would disclose their identity<br>• Downloaded smartphone-specific video tutorials related to oral hygiene maintenance while on orthodontic treatment<br>• Shared two self-photographs (selfies) every week to show their ability to maintain oral hygiene<br>• Each Saturday, the moderator evaluated patients' photographs and level of chat room participation, then published a ranking of the top 5 participants of the week in the chat room<br>o Participants were allowed to share information, pictures, and movies related to oral hygiene and orthodontic treatment
Pretlow et al. [50] (United States)	**Purpose**: Improve weight management<br><br>**Intervention**:<br>• Smartphone App<br>o To guard against unhealthy weight control strategies, the app's provider platform contained range-settable alarm to notify the provider via e-mail if too much weight loss identified through daily weigh-ins checks<br>o Each participant was given with an iPhone 4S, a wireless Bluetooth body weight scale integrated with an app, and a digital food scale<br>o Participants had their mentors' contact details (phone/e-mail) and were able to send them ''eRoom'' (text) messages<br>o Peer support as ''weight loss buddies'', app bulletin boards and app buddy chat<br>o App buddies were matched for age, gender, and weight<br>• Group meetings<br>o Four 2- to 4-hour face-to-face group meetings<br>• Coaching/Mentoring:<br>o Weekly 15-minute call meetings between participants and their coaches or mentors<br>• Other components:

	o Sequential withdrawal from problem foods, snacking, and excessive food amounts at meals<br>o Participants weighted themselves daily<br>o Support to help parents making the family home ‘‘problem food safe,’’ buying no snacks kids<br>o Elimination of snacking<br>Withdrawal from excessive food amounts at home meals
Direito et al. [51] (New Zealand)	**Purpose:** To improve cardiopulmonary fitness in insufficiently active healthy young people<br><br>**Intervention:**<br>• Commercially available apps targeting fitness of the most popular downloaded apps in the Health and Fitness Category of the iTunes New Zealand store available on iTunes and Google Play Store<br>• Two apps included:<br>o An immersive app: Zombies, Run! 5K Training app with a game-themed design embedded with a story where the user is trained to collect supplies and protect a town from zombies<br>o A non-immersive app: Get Running-Couch to 5k app<br>• Both apps:<br>o Consisted of a fully automated 8-week training designed to improve fitness and ability to run 5 km<br>o Provided information on running and technique, audio instructions on how to perform the training components, and tracked and displayed progress throughout the program<br>o Included the ability to work out with music on the device’s library and links to associated websites to interact with other users<br>• Participants were encouraged to use their app 3 times per week and work their way through each of the workouts, but because this was a pragmatic study, access and usage was allowed to vary<br>• No co-interventions, no supplementary modes of delivery, nor usage of prompts (i.e., emails, phone calls, SMS text message) to use the app<br><br>**Control:**<br>• Participants were asked to continue with their usual physical activities. Both apps were provided (free of charge) to participants after trial completion.<br><br>**Intervention and Control:**<br>• Participants were instructed to wear the accelerometer (Actigraph GT1M) on their right hip during waking hours for 7 days after each assessment
