# Supplementary material for: Texting and Mobile Phone App Interventions for Improving Adherence to Preventive Behavior in Adolescents: A Systematic Review
Source: JMIR Mhealth Uhealth. 2017 Apr 19;5(4):e50. doi: 10.2196/mhealth.6837 (PMC5415660; doi:10.2196/mhealth.6837)
Supplement: Multimedia Appendix 5 [file mhealth_v5i4e50_app5.pdf]

Supplemental - Table 4. Outcomes of text message interventions

Source (Purpose)	Main Outcomes	Other Outcomes
Narring et al. [35] (Clinic attendance)	Proportion of unexplained missed appointments, without prior notification<br><br>Intervention vs. Control (95% CI)<br>• All clinics: 16.4% (13.1, 19.8) vs. 20% (16.6, 23.4), *p*=.15<br>• Primary care: 19% (14.5, 23.5) vs. 20.6% (16.1, 25), *p*=.62<br>• Gynecological: 12.9% (6, 19.8) vs. 20.9% (13.2, 28.6), *p*=.13<br>• Mental health: 9.7% (2.7, 16.7) vs. 16.1% (8.5, 23.7), *p*=.23	None
Branson et al. [36] (Clinic attendance)	Attendance rate was calculated as the number of sessions attended divided by the total number of sessions scheduled within 3-month study period<br><br>Intervention vs. Controls, mean±SD:<br>• Attendance rate (unadjusted): 64.9±22.3% vs. 49.3±24.5%, *p*=.026, d=0.67<br>• Attendance rate (adjusted for age, diagnosis, and symptoms severity) remained significantly higher in the intervention arm, *p*=.024 (mean and SD in both arms not reported)	Reminder outcomes:<br>• 88% Received<br>• 12% Not-received: (8% research staff unaware of appointment date/time; and 4% phone broken/lost/ turned off due to lack of minutes)<br><br>Participant satisfaction:<br>• High with majority (82-100%) reporting favorable feedback in 9/10 survey items<br>• Participants suggested two-way communication with therapist for rescheduling and possible delays
Castano et al. [37] (Contraception)	OCPs continuation at 6 months, defined as a patient had taken a pill within the last 7 days prior to a single telephone interview at 6 months after enrollment<br><br>Intervention vs. Controls, n (%):<br>• OCPs users at follow up: 223 (64) vs. 182 (54), *p*=.005<br>• OCPs users at follow up (187 days or less): 76 (75) vs. 53 (54), *p*=.003<br>• OCPs users at follow up (188 days or more): 147 (60) vs. 129 (54), *p*=.16	Intervention vs. Controls, n (%):<br>• No OCPs use interruption for 7 days or more: 203 (59 vs. 162 (48), *p*=.006<br>• Missed no pill during the past month: 136 (39) vs. 92 (27), *p*=.04<br>• OCPs use at last intercourse: 238 (69) vs. 202 (60), *p*=.03<br><br>Text message intervention as a predictor of OCPs continuation at 6 months:<br>• Odds ratio: 1.54 (95%CI, 1.14, 2.1), unadjusted<br>• Odds ratio: 1.44 (95%CI, 1.03, 2), adjusted for age, race, age at coitarche, pregnancy, and past OCP use<br><br>Participant satisfaction:<br>• 85% intervention was helpful<br>• 49% took pill with 1 hour<br>• 91% satisfied with frequency, content, and length of text

		• 49% wanted to continue to receive text messages
Hou et al. [38]<br>(Contraception)	Adherence was measured using:<br>EMD, each day was examined for at least one EMD opening<br>Self-report diary<br><br>Intervention vs. Control using EMD, mean±SD:<br>• Rate of missed pills (all participants): 4.9±3 vs. 4.6±3.5, p=.6<br>• Rate of missed pills (participants completed 3 cycles): 4.1±2.6 vs. 3.7±3.4, p=.67<br>• NS difference in both groups neither at each individual cycle (1, 2 and 3), nor when adherence assessed using self-report diaries<br>• Increased missed pills from week 1 to week 3, in all cycles:<br>o Intervention arm (p=.71)<br>o Controls (p=.007)	Intervention vs. Control:<br>External reminding system (alarms), n (%): 13/36 (36) vs. 26/37 (68), p=.003<br><br>Participant satisfaction:<br>• 66% text messages helped to take pills >50% of the time<br>• 86% would continue or consider continuing text messages<br>• 97% would recommend text to a relative or a friend<br>• 57% were willing to pay <$5 per month to get text reminders<br>• 77% preferred EMD over diary<br>• 41% reported forgetfulness<br><br>Missed pills rate, EMD vs. diary, per cycle:<br>• Overall: 4.7±3.2 vs. 1.2±1.5, $p<.001$<br>• Intervention: 4.9±3 vs. 1.4±1.9, $p<.001$<br>• Control: 4.6±3.5 vs. 1.1±1.2, $p<.001$
Trent et al. [39]<br>(Contraception)	Feasibility measured by eligibility data, and acceptability by enrollment data<br><br>Appointment adherence evaluated by comparing actual clinic visit to schedule visit<br><br>Feasibility and Acceptability:<br>• 110/116 (95%) of approached were eligible for study<br>• 100/110 (91%) of eligible were enrolled in the study<br>• 100% of messages were delivered<br>• Reply rate was 76% to appointment reminders, and 68% to information messages<br><br>On-time visit attendance, Intervention vs. Control:<br>• Cycle 1: 68% vs. 56%<br>• Cycle 2: 68% vs. 62%<br>• Cycle 3: 73% vs. 72%<br><br>Return after scheduled appointment, Intervention vs. Control (days):<br>• Cycle 1: -6 to 18 vs. -7 to 137, p=.03<br>• Cycle 2: -2 to 126 vs. -8 to 127, NS<br>• Cycle 3: -8 to 77 vs. -6 to 28, NS	• Technology access: 92% unlimited text message<br><br>• No confidentiality breach or conflicts reported between patients and parents or partners during study period related to receiving text messages or participating in the study<br><br>• Overall Depo-Provera completion rate: cycle 1 (87%), cycle 2 (77%), and cycle 3 (69%)

Suffoletto et al. [40]<br>(Risk behavior)	Behavioral outcomes were related to: 1) proportion of condom use at last sexual encounter and over past 28 days, and 2) alcohol or drug use before last sex, and unprotected sex and concurrent alcohol use in past 28 days<br><br>Follow up end of study questionnaire overall completion rate, both arms, was 56%<br><br>Condom use with last sexual intercourse, baseline vs. follow up:<br>• Intervention: 20% (95CI, 4-48%) vs. 53% (95CI, 27-79%), *p*=.02<br>• Control: 43% (95CI, 22-66%) vs. 38% (95CI, 18-62%), *p*=.6<br><br>Odds of condom use with last sexual intercourse, Intervention compared to Control: 2.12 (95%CI, 0.52-8.7), independent from baseline status.<br><br>Proportion of always condom use over the past 28 days, baseline vs. follow up:<br>• Intervention: 0% (95%CI, 0-22%) vs. 33% (95%CI, 12-62%), *p*=.01<br>• Control: 24% (95%CI, 8-47%), no change at follow up<br><br>Odds of always condom use over the past 28 days, Intervention compared to Control: 1.32 (95%CI, 0.31-5.71), independent from baseline status.<br><br>Drug or alcohol use before last sex, baseline vs. follow up, n (%):<br>• Intervention: 7 (47) vs. 4 (27), NS<br>• Control: 11 (52) vs. 7 (33), NS<br><br>Any unprotected sex with concurrent alcohol use in past 28 days, baseline vs. follow up, n (%):<br>• Intervention: 10 (67) vs. 4 (27), NS<br>• Control: 8 (38) vs. 2 (10), NS	Weekly queries completion rate:<br>• 70% overall<br>• 88% in week 1<br>• 50% in week 8<br>• 67% in week 12<br><br>About 39% of participants completed all study assessments, and 74% of participants replied to at least 50% of them<br><br>48% of participants reported risky sex behavior during study period over 12-weeks:<br>• 83% of them responded to a goal setting query<br>• 70% were willing to set a goal to not have a risk sex behavior in the coming week, with 29% future risk behavior compared to 100% in those who were not willing to set a goal<br><br>All intervention participants who completed follow up end of study questionnaire reported text messages to be very informative and very useful, with once-weekly interaction being optimal
Cornelius et al. [41]<br>(Risk behavior)	HIV knowledge:<br>• Small increase in HIV knowledge scores from baseline to f/u at 3 month (*p* = .05), higher in older participants 16–18 years<br><br>Attitudes toward condoms:<br>• Scores increased for both genders at immediate posttest, and	Participants responded to daily text messages (total of 91days):<br>• 100% of the time on 20 days<br>• 91%–99% on 46 days<br>• 81%–90% on18 days<br>• 80% on only 7 days

	continued to increase to the 3-month f/u in females, but not males<br>• More positive attitudes in older participants (*p* = .007)<br><br>Perceived HIV risk:<br>• Associated with age (*p* = .03) and time (*p* = .009)<br>• Older participants (16–18 years) were more confident that they can keep themselves from becoming infected with HIV<br><br>HIV risk behaviors:<br>• None of the HIV risk behavior variables showed significant longitudinal change<br>• Fewer unprotected sexual intercourse experiences (vaginal, oral, and/or rectal) over time; baseline (n=31), immediately posttest (n=19) times, and at 3-month follow-up (n=9)	Response time:<br>• Participants responded to the text message boosters within a range of 33.7 to 79.9 minutes, average response time 52.2 min<br>• Facilitators responded to participants' messages within a range of 29.81 to 76.78 minutes, average response time 59.01 min<br><br>Facilitators received an average of 2 additional text messages per week, mainly for response clarification, mobile phone issues and modes of HIV transmission<br><br>Acceptability:<br>• 97% the number of text messages was ''just right''<br>• 89% facilitators answered text message questions timely<br>• 86% text messages did not interfere with their daily activities
Moore et al. [43]<br>(Risk behavior)	Reduction of alcohol consumption from baseline to follow up<br><br>Units of alcohol consumed during the study period, baseline vs. follow up, mean (SD):<br>• Students:<br>o Intervention, 1.9±1.7 vs. 1.5±1.3, NS<br>o Control, 1.6±1.7 vs. 1.5±1.3, NS<br>• Non-students:<br>o Intervention, 1.8±1.4 vs. 1.7±1.8, NS<br>o Control, 1.3±1.1 vs. 1.5±1.2, NS	• Of 87 participants enrolled, 86 responded to at least once to the daily text messages
Haug et al. [44]<br>(Alcohol use)	RSOD in persons with ≥1 RSOD occasion in the last month, baseline vs. follow up, OR (95% CI): 0.66 (0.53-0.83), $p < .001$<br><br>RSOD in persons with >2 RSOD occasions in the last month, baseline vs. follow up, OR (95% CI): 0.76 (0.61-0.94), *p* = .01<br><br>Number of Standard Drinks in a Typical Week, baseline vs. follow up, IRR (95% CI): 0.83 (0.74-0.93), *p* = .002<br><br>Maximum Number of Drinks on an Occasion, baseline vs. follow up, IRR (95% CI): 0.91 (0.83-1.01), *p* = .08<br><br>Alcohol-Related Problems, baseline vs. follow up, OR (95% CI): 0.60 (0.41-0.88), *p* = .009	Individuals dropping out were significantly more likely to:<br>• Be smokers, p = .01<br>• Have more frequent RSOD within the last month, p = .02<br><br>Of the 249 persons with valid data:<br>• 124 (49.8%) read the SMS messages thoroughly<br>• 111 (44.6%) took a short look at the feedback messages<br>• 14 (5.6%) did not read the feedback messages<br><br>196/260 (75.4%) of the program participants received the SMS messages at an appropriate time<br><br>The number of received SMS messages was rated as:<br>• Appropriate by 149/259 (57.5%)

		• More than needed by 92/259 (35.5%)<br>• Less than needed by 18/259 (6.9%)
Haug et al. [45]<br>(Smoking) ^	7-day abstinence, Intervention vs. Control, OR (95% CI):<br>• Total sample: 1.03 (0.59-1.79), *p* = .92<br>• Occasional smokers: 1.56 (0.65-3.75), *p* = .32<br>• Daily smokers: 0.81 (0.36-1.81), *p* = .61<br><br>4-week abstinence, Intervention vs. Control, OR (95% CI):<br>• Total sample: 0.97 (0.50-1.90), *p* = .92<br>• Occasional smokers: 2.06 (0.63-6.78), *p* = .23<br>• Daily smokers: 0.55 (0.17-1.77), *p* = .32<br><br>Number of cigarettes smoked per day at 6 month follow-up, Intervention vs. Control, mean (SD):<br>• Total sample: 7.5 (7.2) vs. 10.0 (7.9), *p* = .002<br>• Occasional smokers: 1.7 (2.4) vs. 2.7 (3.2), p = .02<br>• Daily smokers: 10.2 (7.1) vs. 11.7 (7.7), p = .01<br><br>Quit attempts, Intervention vs. Control, OR (95% CI):<br>• Total sample: 1.18 (0.81-1.72), *p* = .38<br>• Occasional smokers: 2.48 (1.24-4.93), *p* = .01<br>• Daily smokers: 0.95 (0.62-1.46), *p* = .82	No differences in stages of change between the study groups for the total sample, occasional smokers, and daily smokers<br><br>Individuals lost to follow-up, compared to those who did not, were more likely to:<br>• Be daily smokers (81.1% vs. 74.8%; p = .07)<br>• Smoke a higher number of cigarettes per day (11.5 vs. 10.3, p = .048)<br><br>Replies to the weekly SMS text message assessments:<br>• Overall average (SD) number of replies was 6.5 (3.7)<br>• None in 34 participants (9.1%)<br>• 100% in 55 participants (14.8%)<br><br>271/287 (94.4%) participants regularly read the SMS text messages:<br>• 204 (75.3%) read them thoroughly<br>• 67 (24.7%) took a short look at the feedback messages
Bowen et al. [47]<br>(Oral hygiene)	Teeth photographs were evaluated for plaque formation using plainmetry with digital analysis software<br><br>Overall mean reduction in plaque formation in intervention compared to control group was significantly higher, F-test=7.45, *p*<.001<br><br>Mean values of plaque coverage, Intervention vs. Control (95%CI):<br>• T0: 0.36 (0.31-0.42) vs. 0.43 (0.38-0.48), NS<br>• T1 0.24 (0.18-0.29) vs. 0.44 (0.39-0.49), *p*<.05<br>• T2: 0.22 (0.16-0.28) vs. 0.58 (0.52-0.64), *p*<.05	Inter-rater or inter-judge reliability was:<br>• 0.952 for tooth size analysis, *p*<.001<br>• 0.972 for plaque analysis, *p*<.001
Lau et al. [48]<br>(Physical activity)	Self-report of PA in previous 7 days measured by PAQC and motivational readiness measured by SMR Questionnaire<br><br>Self-report of PA (PAQC), baseline vs. follow up, mean (SD):<br>• Intervention: 1.85±0.27 vs. 2.03±0.52, *p*<.05<br>• Control: 1.77±0.36 vs. 1.85±0.63, NS	Internet program/text messages frequency/duration exposure rate, and customized satisfaction questionnaire<br><br>Participant satisfaction:<br>• 81% would like to continue receiving text messages<br>• 59% would recommend Internet PA program and text messages for a their friends

	Motivational readiness (SMR), baseline vs. follow up, median:<br>• Intervention: 1 vs. 2, p<.05<br>• Control: 1 vs. 1, NS<br><br>Number of text messages read correlated with changes in SMR (r=0.47, p=0.009), and persisted when controlled for Internet PA program exposure (r=0.46, *p*=.01)	Internet PA program exposure:<br>• 100% logged in at least once, and 66% did 2 or more times<br>• Mean weekly login rate 0.5/person<br>• Mean total visit time 30 min/person, with 3.75 min/visit<br><br>Text messages exposure:<br>• 79% participants read average of 1.3 message/person/week
Abraham et al. [49] (Weight management)	Feasibility of adapting and using internet-based nutritional program and text messages for follow up as an intervention<br><br>• Internet-intervention:<br>o All participants logged in to the system<br>o 14/16 (87.5%) read the curriculum<br>o 10/14 (71%) completed all lessons<br>o 400 messages sent to 16 participants<br>o 15 preferred Whatsapp to get messages and 1 preferred email<br>o 78.3% response rate to dietary goals<br>o 77.5% response rate to exercise goals<br>o Research assistant spent 2 hours weekly on average to send personalized messages to all participants	• 93.5% of all participants found obesity consultations with a pediatrician useful<br><br>• sLMP-intervention:<br>o Obesity counseling with the nutritionist was consider to be useful or very useful by all parent and 94% participants<br>o 94% completed all sessions<br>o Nutritional counseling show-up on time: 100% (T0), 81.3% (T2), 62.5% (T4) and 0% (T12) of all participants<br><br>• Secondary outcomes:<br>o Borderline significant decrease in systolic (*p*=.05) and diastolic (p = .047) blood pressure at T12 and T24 compared to T0, only in sLMP group<br>o No significant changes in body fat, BMI, physical activity in 3 study groups
Sachse et al. [52] (Sun protection)	Feasibility and acceptability of text-messages, and sun-protection knowledge and behavior<br><br>Feasibility and acceptability of text messages (wk-8):<br>• 19/26 (73%) participants complete phone interview at week 8<br>• 18/19 (95%), read sun-protection text messages daily<br>• 18/19 (95%), text was helpful to remember content of training<br>• 10/19 (53%), increased their application of sunscreen<br>• 11/19 (58%), changed their sun-protective behavior when the text indicated UV-index was high<br><br>Sun-protection knowledge Q (wk-4), baseline vs. follow up:<br>• 16% vs. 75%, understanding of the meaning of UV-index<br>• 0% vs. 37%, naming at least 3 of the ABCDE mnemonic for skin self-exam	

	• 26% vs. 47%, knowing that it takes hours to recognize sunburns	
Matheson et al. [53] (Vaccination)	HPV vaccination series completion rate, and in relation to due date<br><br>Intervention vs. historical control, n (%):<br>• HPV 2: 27 (73) vs. 78 (27), *p*<.001<br>• HPV 2 on time: 14 (38) vs. 59 (21), *p*=.04<br>• HPV 3: 6 (16) vs. 14 (5), *p*=.02<br>• HPV 3 on time: 5 (14) vs. 7 (3), *p*=.007<br><br>Intervention vs. interested group, n (%):<br>• HPV 2: 27 (73) vs. 14 (33), *p*<.001<br>• HPV 2 on time: 14 (38) vs. 9 (21), *p*=.14<br>• HPV 3: 6 (16) vs. 0 (0), *p*=.008<br>• HPV 3 on time: 5 (14) vs. 0 (0), *p*=.02	

* Statistically significant *p*<0.05
ABCDE: asymmetry, border, color, diameter, and evolution
BMI: body mass index
CI: confidence interval
EMD: electronic monitoring device
HPV: human papilloma virus
IQR: inter-quartile range
IRR: Incidence Risk Ratio
N: number
NS: non significant
OCPs: oral contraceptive pills
OR: odds ratio
PA: physical activity
PAQC: physical activity questionnaire for older children
Q: questionnaire
SD: standard deviation
sLMP: simplified lifestyle modification program
SMR: stages of motivational readiness
SOC: standard of care
STIs: sexually transmitted infections
UV: ultra-violet
^ All data presented from the study intention-to-treat analysis of the outcomes
