# Supplementary material for: Texting and Mobile Phone App Interventions for Improving Adherence to Preventive Behavior in Adolescents: A Systematic Review
Source: JMIR Mhealth Uhealth. 2017 Apr 19;5(4):e50. doi: 10.2196/mhealth.6837 (PMC5415660; doi:10.2196/mhealth.6837)
Supplement: Multimedia Appendix 6 [file mhealth_v5i4e50_app6.pdf]

Supplemental - Table 5. Outcomes of mobile phone app interventions

Source (Purpose)	Main Outcomes	Other Outcomes
Lopez et al. [42]<br>(Risk behavior)	Non-regular sex partners, baseline (first survey) vs. 6 mon f/u (second survey), n (%):<br>• Men: 12/27 (44%) vs. 13/27 (48%), NS<br>• Women: 8/31 (26%) vs. 9/31 (29%), NS<br><br>Condom use with non-regular partner during last sexual intercourse, baseline vs. 6 mon f/u, n (%): 7/58 (12%) vs. 6/58 (10%), NS<br><br>Sexually transmitted disease (STD) knowledge, baseline vs. 6 mon f/u, % (n):<br><br>A. Foul-smelling discharge is a symptom that can affect, *p* = .04:<br>• Both men and women: 69% (40/58) vs. 86% (50/58)<br>• Only men or only women: 26% (15/58) vs. 9% (5/58)<br>• Neither: 5% (3/58) vs. 5% (3/58)<br><br>B. Anal irritation or discharge as a symptom of STDs:<br>• Not true: 41% (24/58) vs. 31% (18/58), NS<br>• True but only in men or only women: 19% (11/58) vs. 12% (7/58), NS<br><br>C. HIV transmission:<br>• Sharing food: 12% (7/58) vs. 7% (4/58), NS<br>• Mother to newborn by breastfeeding: 19% (11/58) vs. 17% (10/58), NS	Alcohol use ≥1/week in 6 months, baseline vs. 6 mon f/u, % (n): 26% (15/58) vs. 31% (18/58), NS<br><br>Psychoactive substance use ≥1 time, baseline vs. 6 mon f/u, % (n): 41% (24/58) vs. 38% (22/58), NS<br><br>Number of consultations, n (%):<br>• 1 to 3 times: 48/58 (83%)<br>• 4 or more: 9/58 (16%)<br>• 29 times: 1 (2%)<br><br>Satisfaction with DoctorChat Mobile app:<br>• Overall experiences:<br>o Excellent (26.8%)<br>o Good (50.0%)<br>o Fair (15.5%)<br>o Bad, (7.7%)<br>• Ease of use:<br>o Easy or very easy (69.7%)<br>o Difficult or not so easy (30.3%)<br>• Having access to a mobile-based tele-consultation tool on sexual health to be important or very important (92.3%)
Zotti et al. [46]<br>(Oral hygiene)	Every 3 months, participants in both groups were examined in-person and evaluated for GI and PI scores, and number of WS<br><br>GI, Intervention vs. Control, mean (SD):<br>• T0: 1.2±0.7 vs. 1.2±0.7, NS<br>• T1: 1.1±0.5 vs. 1.4±0.6, NS<br>• T2: 0.99±0.5 vs. 1.3±0.6, *p*<.05<br>• T3: 0.87±0.4 vs. 1.4±0.6, *p*<.001<br>• T4: 0.67±0.4 vs. 1.4±0.6, *p*<.0001<br><br>PI, Intervention vs. Control, mean (SD):<br>• T0: 0.4±0.3 vs. 0.5±0.3, NS	No statistically significant difference observed in the frequency of new caries across intervention and control groups during study period of 12-month<br><br>All participants in intervention group uploaded their selfies every week throughout the study<br><br>Some participants uploaded additional content, including pictures and video tutorials related to oral hygiene and orthodontic treatment<br><br>A community of peers sharing their experience was successfully created among the intervention participants

	• T1: 1.7±0.4 vs. 1.7±0.3, NS<br>• T2: 1.5±0.3 vs. 1.8±0.5, *p*<.01<br>• T3: 1.3±0.4 vs. 1.9±0.4, *p*<.0001<br>• T4: 1.1±0.5 vs. 1.8±0.5, *p*<.0001<br><br>N of patients with visible WS, Intervention vs. Control:<br>• T0: 4 vs. 5, NS<br>• T1: 4 vs. 6, NS<br>• T2: 4 vs. 8, NS<br>• T3: 5 vs. 13, *p*<.05<br>• T4: 7 vs. 16, *p*<.05	
Pretlow et al. [50]<br>(Weight management)	Change in % over-BMI:<br>• Weight loss average was 7.1 %over-BMI (baseline to end of study), 0.051 per day (*p*<.01)<br>• More weight loss was seen in: males (standardized estimate = 0.71; *p*<.01), younger patients (standardized estimate = 0.42; *p*=.04)<br><br>Self-management/Adherence:<br>• Improvement in the following (baseline vs. end of the study): better control of eating (*p*<.01), higher self-esteem (p<0.01), and less turning to food at stress (*p*<.01)<br>• Participants with better weight loss:<br>o Addiction model did not affect their guilt about their weight (standardized estimate = 0.59; *p*<.01)<br>o Had high program compliance as reported by their mentors (standardized estimate = 0.68; *p*<.01)<br>o 48.1% indicated that calling obesity an addiction made their guilt about their weight worse, more in girls (66.7 vs. 11.1%, *p*<.01)	Usability/Acceptability:<br>• Average satisfaction 3.1 (5 being most helpful)<br>• 25/27 (89%) completed the program were able to identify one or more specific problem foods<br>• 70% completely eliminated and 30% reduced frequency of their snacking<br>• 26/27 (96%) reduced amounts of home meals, average 51.1% less from baseline
Direito et al. [51]<br>(Physical activity)	In pre-specified per-protocol analyses with the app being used 3 times/week), Time to complete 1-mile walk/run, adjusted mean difference in seconds (95% CI):<br>• Non-immersive app vs. control: –79.39 (–133.01, –25.77), *p* = .003<br>• Immersive app vs. control: –55.29 (–111.46, 0.88), *p* = .06<br><br>Intervention "immersive" vs. Control, adjusted difference (95% CI):<br>• Time to complete 1-mile walk/run (sec): –28.36 (–66.54, 9.82)<br>• PAQ-A: 0.14 (–0.26, 0.54)<br>• PACES: –0.10 (–0.33, 0.13)<br>• PNSES: –0.08 (–0.46, 0.31)<br>• PASES: –0.02 (–0.24, 0.19)	No adverse events were deemed related to the study intervention<br><br>Intervention apps use:<br>• 2 times per week in 10/32 (31%)<br>• 3 times per week in 10/32 (31%)<br>• 1 time per week in 8/32 (25%)<br><br>No differences were evident on timing of use (i.e., weekday, weekend, morning, afternoon, evening)<br><br>Intervention apps were used:<br>• Mostly outdoors (e.g., street, park vs. gym, home treadmill)

	• Daily time spent in sedentary activities (min): –10.94 (–69.83, 48)<br>• Daily time spent in MVPA (min): 1.74 (–11.45, 14.93)<br><br>Intervention "non-immersive" vs. Control, adjusted difference (95% CI):<br>• Time to complete 1-mile walk/run (sec): –24.67 (–63.51, 14.18)<br>• PAQ-A: 0.23 (–0.18, 0.64)<br>• PACES: –0.17 (–0.40, 0.06)<br>• PNSES: 0.01 (–0.38, 0.40)<br>• PASES: 0.04 (–0.18, 0.26)<br>• Daily time spent in sedentary activities (min): 3.95 (–56.26, 64.16)<br>• Daily time spent in MVPA (min): –1.82 (–16.00, 12.36)	• With friend (n=7), family (n=9), or alone (n=13)<br><br>The majority of participants (21/32, 66%) had no prior experience of using their smartphone for PA purposes<br><br>Overall, 81% (26/32) were interested in trying different PA-promoting apps in the future

App: Application
BMI: Body mass index;
GI: Gingival index
MVPA: moderate-to-vigorous physical activity
PACES: Physical Activity Enjoyment Scale
PAQ-A: Physical Activity Questionnaire for Adolescents
PASES: Physical Activity Self-Efficacy Scale
PI: Plaque index
PNSES: Psychological Need Satisfaction in Exercise Scale
SD: Standard deviation
SOC: Standard of care
WS: White spots
GI measurement score (0-3): 0 being normal gingiva and 3 having severe inflammation and edema, with spontaneous bleeding
PI measurement score (0-3): 0 being best with no plaques and 3 having plaque covering more than half of the surface
WS measurement score: (0-3): 0 being no visible white spots, and 3 having visible white spots requiring restoration
